# A Novel Machine Learning Strategy for the Prediction of Antihypertensive Peptides Derived from Food with High Efficiency

**DOI:** 10.3390/foods10030550

**Published:** 2021-03-06

**Authors:** Liyang Wang, Dantong Niu, Xiaoya Wang, Jabir Khan, Qun Shen, Yong Xue

**Affiliations:** 1 College of Food Science and Nutritional Engineering, China Agricultural University, Beijing 100083, China; 18259800533@163.com (L.W.); 15384665858@163.com (X.W.); Kjabir135@gmail.com (J.K.); shenqun@cau.edu.cn (Q.S.); 2 College of Information and Electrical Engineering, China Agricultural University, Beijing 100083, China; niudantong.88@gmail.com

**Keywords:** antihypertensive peptides, XGBoost algorithm, peptide–protein technology, milk protein, high throughput, high efficiency

## Abstract

Strategies to screen antihypertensive peptides with high throughput and rapid speed will doubtlessly contribute to the treatment of hypertension. Food-derived antihypertensive peptides can reduce blood pressure without side effects. In the present study, a novel model based on the eXtreme Gradient Boosting (XGBoost) algorithm was developed and compared with the dominating machine learning models. To further reflect on the reliability of the method in a real situation, the optimized XGBoost model was utilized to predict the antihypertensive degree of the k-mer peptides cutting from six key proteins in bovine milk, and the peptide–protein docking technology was introduced to verify the findings. The results showed that the XGBoost model achieved outstanding performance, with an accuracy of 86.50% and area under the receiver operating characteristic curve of 94.11%, which were better than the other models. Using the XGBoost model, the prediction of antihypertensive peptides derived from milk protein was consistent with the peptide–protein docking results, and was more efficient. Our results indicate that using the XGBoost algorithm as a novel auxiliary tool is feasible to screen for antihypertensive peptides derived from food, with high throughput and high efficiency.

## 1. Introduction

Hypertension, also known as cardiovascular syndrome, is a modifiable risk factor responsible for a high burden of disability and death [1]. In the early stage, hypertension patients may have no obvious symptoms, but long-term high blood pressure will burden the arteries and cause ventricular hypertrophy, endangering the critical organs such as the heart, brain, and kidney [2,3]. Currently, although drug intervention is the conventional treatment, there is increasing evidence to indicate that non-pharmacologic strategies, such as dietary modifications, could have a considerable effect in the prevention and treatment of hypertension [4,5].

Most of the antihypertensive drugs belong to diuretics, adrenergic receptor blockers, calcium channel blockers, and angiotensin converting enzyme (ACE) inhibitors. Despite having satisfactory antihypertensive effects, they do have documented side effects. On the contrary, food-derived antihypertensive peptides, a specific class of micro-molecule peptides, can reduce blood pressure with many attractive advantages. For example, they have impressive performance while causing no excessive reduction in blood pressure. Moreover, their properties of have no toxicity, side effects, or adverse reactions. Therefore, the discovery and verification of these peptides have brought to the fore the current focus on such research [6,7]. ACE, as a zinc-containing dipeptide carboxypeptidase, plays a vital role in the regulatory mechanism of blood pressure, including the renin angiotensin system and kallikrein kinin system, so that inhibiting ACE activity is considered as a key measure to treat hypertension. Currently, a large numbers of effective and specific ACE inhibitors, including some bioactive peptides derived from food, are used for the treatments of hypertension [8]. The traditional screening methods for ACE-inhibitory peptides mainly depend on in vitro or/and in vivo experiments, which are time-consuming and expensive [9]. Thus, it is necessary to establish a screening method for antihypertensive peptides with a high throughput and speed.

With the development of artificial intelligence technology, machine learning has been applied in the field of drug screening, which could shorten the experiment period and make a great achievement to a large degree by studying the characteristics of known drug molecules. There have been many studies on the research of ACE-inhibitory peptides by employing the quantitative structure activity relationship (QSAR) technique and traditional machine learning models [10,11]. Furthermore, machine learning strategies (including deep learning) were also adopted to screen other targets, such as dipeptidyl peptidase-4 (DPP-IV) inhibitors and anticancer peptide [12,13,14]. The feature extraction of the primary structure is very important for the activity prediction of peptides. Chou et al. [15,16] proposed a method named PseAAC (pseudo amino acid composition) to represent the features of amino acid composition that combine their physical and chemical properties, and this method has been widely used in bioinformatics of protein function. Therefore, this study was proposed by using PseAAC to exploit the features extracted from ACE-inhibitory peptides (positive samples) and the peptides (negative samples) randomly selected from the UniProt library, and then was designed using a parameter-optimized eXtreme Gradient Boosting (XGBoost) machine learning model to train and predict. Though the XGBoost model has been widely used in many fields because of its superior structure [17,18], such utilizations have been rarely found in the research of drug screening.

In this study, three public databases of ACE-inhibitory peptides were trained using three dominating machine learning methods and the XGBoost model. Through 5-fold cross-validation, it was found that the XGBoost model demonstrates the best performance in the screening task of ACE-inhibitory peptides. A negative association between milk consumption and blood pressure has been reported in human studies, likely due to some peptides derived from milk that were found to have antihypertensive effects [6,19]. In order to verify the generalization ability of this method, six proteins with an unambiguous structure in bovine milk were selected initially as row sequences to get k-mer peptides using the cycle cutting algorithm. After predicting by the XGBoost model, the candidate inhibitory and non-inhibitory peptides were further confirmed by using the peptide–protein docking technology.

## 2. Method and Materials

**Figure 1:**
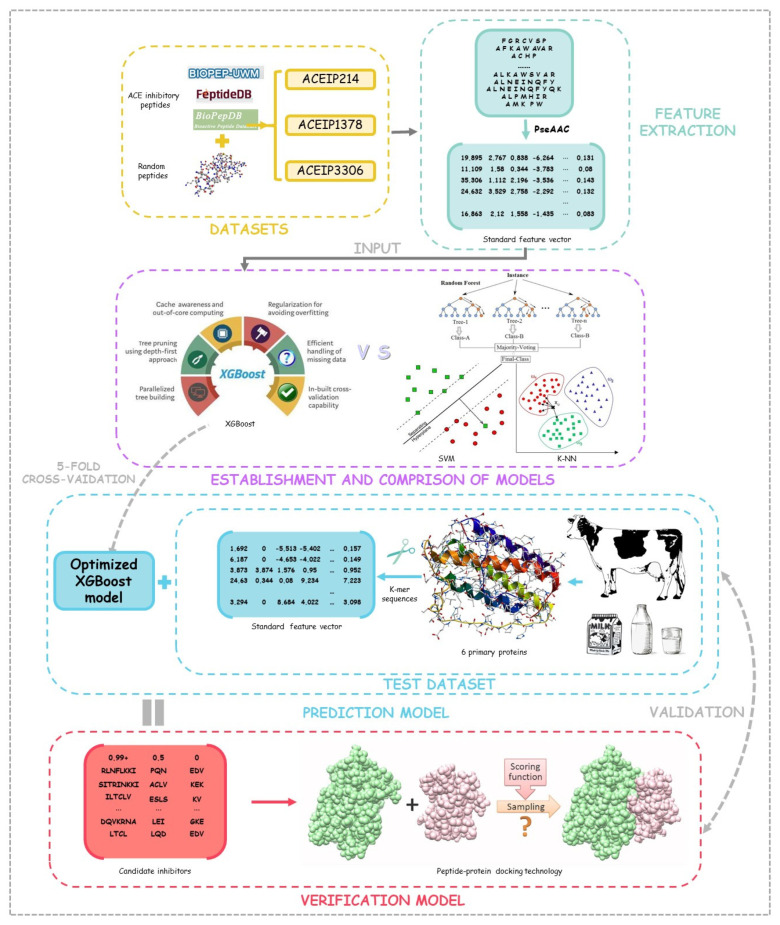
The flow chat of the data analysis.

In this research, the computer was configured with a CPU Intel Core I7-6700HQ, 3.5 GHz, 4 GB of memory, and the experimental programming was implemented in Python 3.8. The composition of all 20 amino acids in the peptides were counted and compared in our datasets, and then the benchmark feature vectors obtained via the PseAAC algorithm were input into the models. Through cross-validation, the performance of the XGBoost algorithm and some other algorithms were compared, and the optimal model was selected out for subsequent testing. During the testing process, the k-mer algorithm was utilized to cut the primary six proteins in the bovine milk, and short peptides with different lengths were used as the test dataset, and then the probability of ACE-inhibitory ability obtained by our optimized model was generated as the prediction results. In order to show the reliability of our method, the peptide–protein docking technology was exploited to verify the prediction results. The workflow of the study is shown in Figure 1. 

### 2.1. Training Data and Test Data 

In this study, the databases of ACE inhibitory peptides for training were obtained from three public databases, including BIOPEP-UWM [20], FeptideDB [21], and BioPepDB [22], according to the functional annotation. After screening, 107, 689, and 1653 peptide sequences, which were unambiguously tagged as antihypertensive peptides, were selected as the positive samples. It is worth noting that those antihypertensive peptides with the same sequence were only reserved in one of these databases. According to the same strategy employed in previous studies [23,24], this study randomly selected the peptides from the UniProt library as the negative samples, which met Equations (1) and (2) and had a similar average sequence length with the positive samples. The positive samples from the three public databases and their corresponding negative samples composed our new datasets, named ACEIP214, ACEIP1378, and ACEIP3306, respectively.

(1) $$I=I^{+}ØI^{-}$$

(2) $$\emptyset =I^{+}\cap I^{-}$$

where $I^{+}$ represents the positive samples of the antihypertensive peptide, $I^{-}$ represents the negative samples, and I represents the whole dataset. There was no overlap between $I^{+}$ and $I^{-}$.

Furthermore, with the aim to test the prediction ability of the models in a real situation, six key proteins in bovine milk were selected from the UniProt protein library and their specific information is shown in Table 1. Using the cycle cutting algorithm, totally more than 10,000 k-mer peptides (k = 2, 3, …, 9) [25] were generated from the six proteins as the test dataset.

### 2.2. Representation of Peptide Sequence Feature

As widely recognized, the functions of protein largely depend on the 3D structure and some key residues called the reaction center. The structure and the key residues of a protein are fundamentally based on the amino acids sequence, indicating that it is possible to infer the function of the peptide from the amino acid sequence. Additionally, extracting the features of peptides attracts wide interest since the rise of machine leaning technology and several methods have been put up, such as the AAC (amino acid composition) [26], PseAAC (modified algorithm based on the classic AAC), and the binary profile of the patterns. In the present study, the type II PseAAC was adopted as the feature extraction method of the peptides.

When encoding the peptide sequence with PseAAC, each peptide can be represented by a vector with 20+iλ dimensions, where i denotes the number of properties of the amino acid taken into consideration and λ is a coefficient that determines the distance of the interacted amino acids (if λ equals 1, only the interaction between the adjacent amino acids would be considered). Thus, PseAAC is a kind of comprehensive encoding method that includes information of both the internal composition and external interaction of the amino acids. In our study, the amino acid properties we chose were hydrophobicity, hydrophilicity, mass, pK_1_ (α-CO_2_H), pK_2_ (NH_3_), and pI (at 25 °C). λ was set to 1, and the weight factor, ω, which is used to adjust the degree of influence of the amino acid sequence order information on the classification system, was set to 0.05.

### 2.3. Machine Learning Algorithms

In this paper, the feature vectors of the input models were the amino acid sequence feature (26 dimensions) extracted by PseAAC, which was used to extract the primary structural, physical, and chemical features of the samples. The following parameter-optimized models were performed with a binary classification task and were also employed to make comparisons to each other. In the model optimization process, the grid search strategy was employed for the adjustment of the parameter. The introduction and parameter settings of the specific models are as follows. 

#### 2.3.1. Extreme Gradient Boosting

XGBoost belongs to one of the boosting algorithms, the idea of which is to integrate many weak classifiers together to form a strong classifier. As a boosting tree model, XGBoost is a powerful classifier composed of many single tree models. The summary of the prediction values generated by each individual in the k-tree (k is the number of single trees) were used in the XGBoost model. During each iteration of the prediction, a new function would be introduced to minimize the objective function as much as possible. Except for the linear classifiers employed in the XGBoost model, a regular term is also added to the cost function to control the complexity of the model. Moreover, in contrast with other ensemble learning methods that only use the information of the first-order derivative, the first- and second-order derivatives were considered together, and the second-order Taylor expansion of the cost function was executed in the XGBoost model. It is worth noting that the XGBoost model has been widely used in various fields due to the superiority of its principle [27,28]. The XGboost library in Python was selected for experiments, and the key parameters learning_rate = 0.01, n_estimators = 1000, max_depth = 4, min_child_weight = 1, gamma = 0, and subsample = 0.8 were set.

#### 2.3.2. Support Vector Machine (SVM)

The SVM model is a kind of classic machine learning algorithm, which belongs to the supervised learning algorithm to solve the two or multi-classification problem. Furthermore, the SVM has been employed to solve nonlinear problems with the introduction of the kernel function. The basic principle of the SVM model is to find the best separation hyperplane in the feature space, so that the intervals between the positive and negative samples can be maximized. Currently, the SVM model has been utilized for peptide prediction as well [14,24]. A non-linear SVM classifier was adopted in this study, and the RBF kernel function was selected. The penalty coefficient C was set to 1.0, and the gamma was set to 0.001.

#### 2.3.3. Random Forest (RF)

The RF model is a typical algorithm of Bagging-type ensemble learning, which integrates multiple weak classifiers to improve the overall accuracy and generalization ability. Though the RF model has been adopted in several research to recognize the bioactive peptides, it undermines when dealing with unbalanced data of the peptides with a high-dimension characteristic [29,30]. The key parameters of the RF classifier were set to n_estimators = 80, max_depth = 13, min_samples_split = 150, min_samples_leaf = 15, max_features = 7, oob_score = True, and random_state = 10.

#### 2.3.4. K-Nearest Neighbor (K-NN)

As one of the classic algorithms in machine learning, the K-NN classifier is widely adopted in various research topics because of its relatively simple principle and training process. It calculates the distance between the new data and the training data, and then selects k (k ≥ 1) closest neighbors to make classification or regression. The K-NN model has been applied in protein recognition [31]. However, it is inevitable to face the low interpretability and prediction accuracy of rare categories when the number of samples was unbalanced. This paper chose a supervised learning K-NN classifier, and it was found that the effect was best when K = 5 through multiple adjustments.

### 2.4. Performance Evaluation of Models 

Firstly, a 5-fold cross-validation in the single dataset was executed in this study, and the results were displayed by the commonly used evaluation criteria, including Accuracy (Acc), Sensitivity (Sens), Specificity (Spec), and Precision (Prec), and the area under the receiver operating characteristic curve (AUC). In order to further verify the generalization ability of the model, in addition to experimenting in a single dataset, the study also chose the dataset ACEIP3306 as the training set (considering factors such as the amount of data and the model’s best performance in this dataset), and then employed ACEIP214 and ACEIP1378 as the test dataset. The above process was repeated 5 times and the AUC values of the best model were counted. Additionally, the three datasets were merged into a total dataset, and on this basis, a 5-fold cross-validation was performed, and the AUC value of the best model was counted. The threshold of classification was set to 0.5 in the present study; that is, when the probability of a peptide predicted by the model was higher than 0.5; it was judged as a positive one. Meanwhile, it cannot be ignored that the same threshold was used to compare among the different methods.

(3) $$Acc=\frac{TN+TP}{TN+TP+FN+FP}$$

(4) $$Sens=\frac{TP}{TP+FN}$$

(5) $$Spec=\frac{TN}{TN+FP}$$

(6) $$Prec=\frac{TP}{TP+FP}$$

where *TN* represents the true negative number, *TP* signifies the true positive number, *FN* denotes the false negative number, and *FP* stands for the false negative number.

### 2.5. Prediction Model and Peptide–Protein Docking Verification

To test the prediction ability of our ACE-inhibitory peptide model in the real situation, the optimal model was utilized to do high-throughput and rapid screening of the test dataset (over 10,000 peptides cutting from the key proteins rich in bovine milk). The experiments were performed in parallel three times (the optimized model was trained firstly and then tested, and all of the process was repeated three times), and the possibility of a positive peptide was calculated. When the possibility of one peptide is over 99.00% for all the three times, the peptide can be recognized as the one with anti-hypertensive activity in our study. Furthermore, to discover the difference between the positive and negative peptide predicted in the present study, two groups of peptides with a possibility of 0.00% and 50.00% were both selected as the negative groups. The screening results of our model were further verified via peptide–protein docking technology. With help of virtual screening technology, discovering new inhibitors is becoming a common practice in modern drug discovery [32]. Furthermore, the structure-based virtual screening approach is widely employed in this field due to its cost-effective and time-saving advantages. In our study, virtual screening was applied to validate the prediction results of our model. HPEPDOCK Server was selected to carry out the virtual screening task due to its outstanding performance and accurate result [33,34,35]. Considering the fact that the reaction center of ACE is clearly known, it is reasonable to judge the docking result by the docked free energy (measured as the docking scores). Theoretically, peptides that are fixed to the pocket of the reaction center with lower affinity energy are more likely to be the inhibitors and vice versa.

## 3. Results

### 3.1. Distribution of Amino Acids in the Datasets

**Figure 2:**
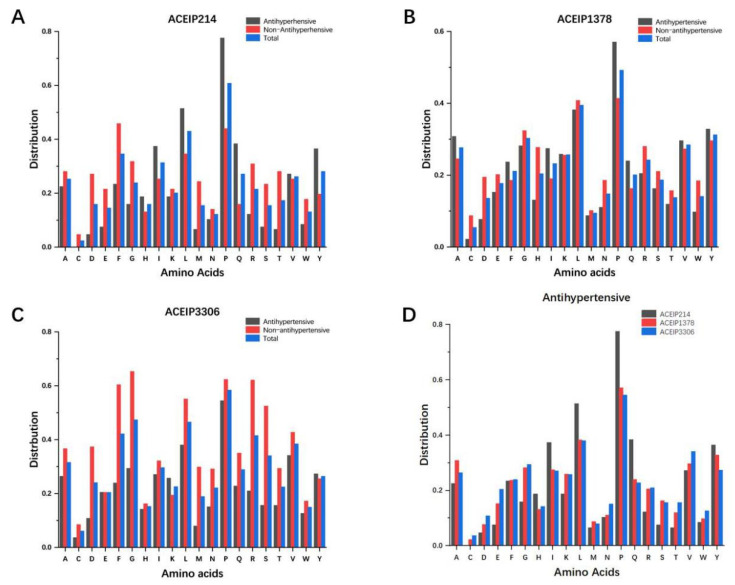
The frequency distribution of the various amino acids in peptides from the three datasets: ACEIP214 (**A**), ACEIP1378 (**B**), ACEIP 3306 (**C**), and comparison of the amino acid distributions of the positive samples in the three datasets (**D**).

The research counted and compared the amino acid distribution of the positive, negative, and total samples in our three benchmark datasets, respectively (Figure 2). Studies have shown that the distribution of amino acid residues affects the biological activity of peptides [14,23]. From the frequency of amino acids in the positive samples, the distribution of 20 amino acids is relatively consistent among the three datasets. It is obvious that *Pro* and *Leu* appeared frequently in ACE-inhibitory peptides, while *Cys*, *Met*, and *Trp* were rare [36]. However, it is undeniable that the amino acid distributions of the three datasets have dissimilarities, too. For example, the proportion of *Pro* and *Leu* in ACEIP214 was significantly higher than that in ACEIP1378 and ACEIP3306. 

### 3.2. Results of XGBoost Model

**Figure 3:**
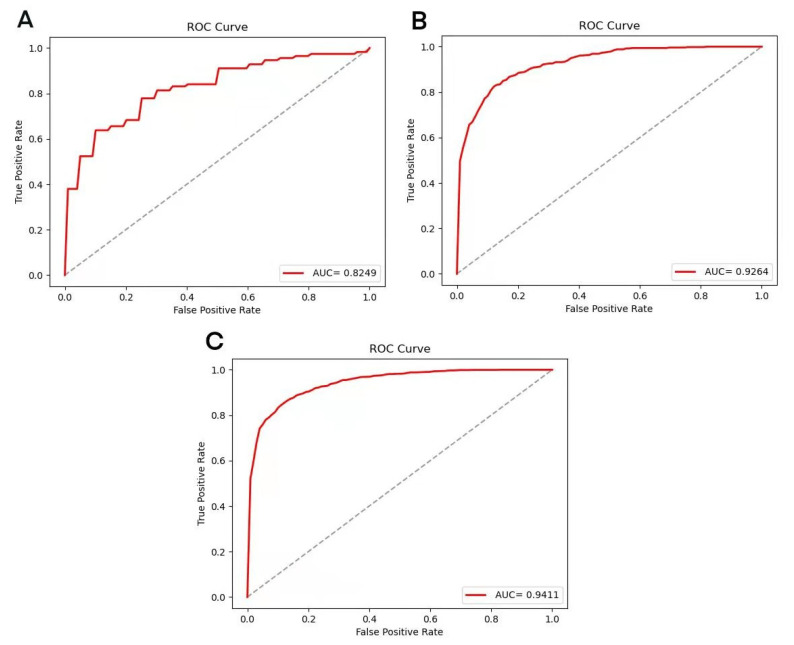
Performance of the XGBoost model on datasets ACEIP214 (**A**), ACEIP1378 (**B**), and ACEIP3306 (**C**). ROC, the receiver operating characteristic curve; AUC, the area under the receiver operating characteristic curve.

The XGBoost model was adopted to execute 5-fold cross-validation based on the three datasets ACEIP214, ACEIP1378, and ACEIP3306 (Table 2). The best performance of the XGBoost model was achieved in ACEIP3306, with a mean accuracy of 86.50%, average sensitivity of 86.08%, average specificity of 86.92%, and average precision of 86.85%, which reflected the excellent performance and strong generalization ability of the XGBoost algorithm. In order to comprehensively display the performance of the model, the receiver operating characteristic curve (ROC) and AUC were introduced (Figure 3). It was obvious that significant differences existed among the AUC value of the different datasets (ACEIP3306 had the largest AUC of 94.11%, followed by the 92.64% for ACEIP1378 and 82.49% for ACEIP214). 

In addition, the results between the different datasets (ACEIP3306 for training and the rest of the datasets for testing) and performance on the total dataset are shown in Table 3.

### 3.3. Results of Other Models

**Figure 4:**
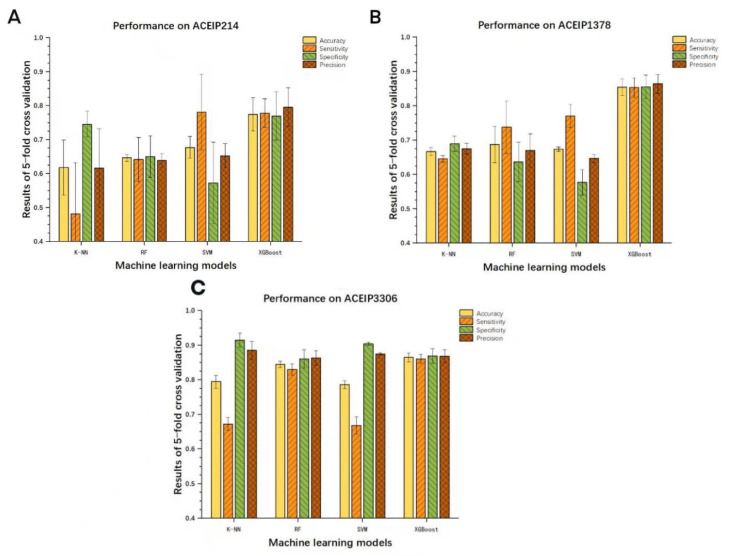
Comparisons and evaluations of the different machine learning models on datasets ACEIP214 (**A**), ACEIP1378 (**B**), and ACEIP3306 (**C**).

**Figure 5:**
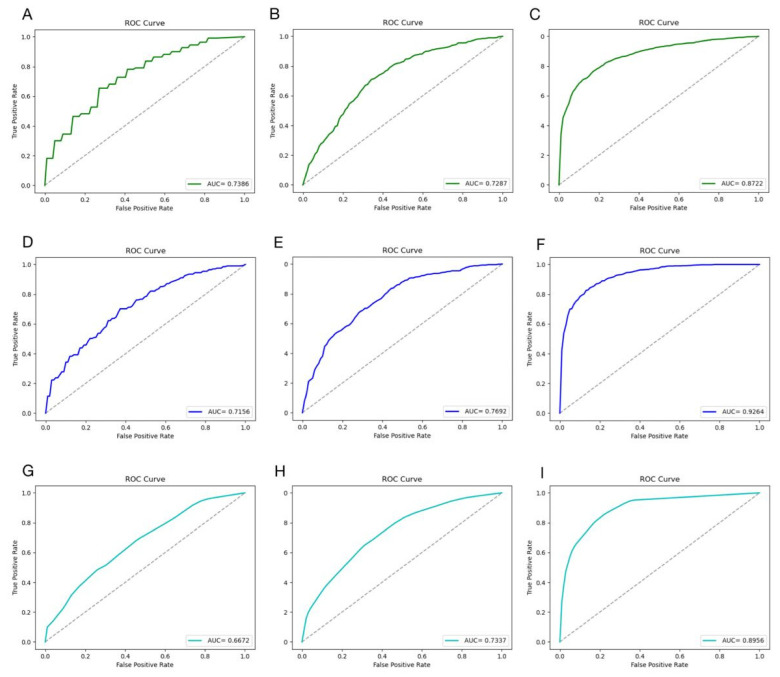
The performance of the machine learning algorithms on different datasets. The performance of the SVM model on ACEIP214 (**A**), ACEIP1378 (**B**), and ACEIP3306 (**C**); the performance of the RF model on ACEIP214 (**D**), ACEIP1378 (**E**), and ACEIP3306 (**F**); and the performance of the K-NN model on ACEIP214 (**G**), ACEIP1378 (**H**), and ACEIP3306 (**I**). ROC, the receiver operating characteristic curve; AUC, the area under the receiver operating characteristic curve.

The work further constructed the models with other machine learning models and made a comparison with the XGBoost algorithm (Figure 4). Our results showed that the XGBoost model exhibited a remarkable ability when compared with the other algorithms. However, it needs to be admitted that the RF algorithm performed well on the dataset ACEIP3306, only slightly lower than the XGBoost algorithm, and better than the SVM and K-NN algorithms. Additionally, as the amount of data increases, two interesting trends could be seen. First, it was found that the overall performance of each model was more superior from the evaluation indicators, and the other was that each model was more “robust” from the 95% CIs. The ROC curves and AUC values of the other three classic machine learning methods on different datasets are showed in Figure 5. Comparing with the performance of the XGBoost model, all three of the classic machine learning algorithms had lower values of AUC based on the three datasets, supporting the outstanding generalization and superior ability of the XGBoost model for screening ACE-inhibitory peptides. 

### 3.4. Prediction Model and Peptide–Protein Docking Verification

**Figure 6:**
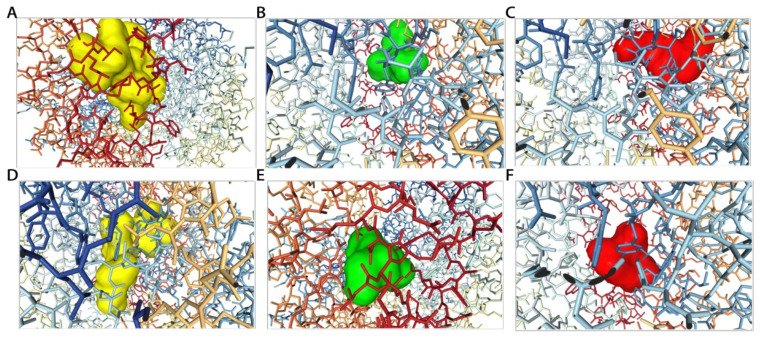
The 3D structural diagrams of the peptide–protein docking of RLNFLKKI (**A**), PQN (**B**), EDV (**C**), SITRNKKI (**D**), ACLV (**E**), and KEK (**F**).

Over 10,000 peptides were obtained by randomly cutting the six key proteins in bovine milk, and the study further chose the XGBoost model based on ACEIP3306 to predict ACE-inhibitory peptides. In the present study, the group a with a probability value of prediction over 0.99, for 3 times (training once and testing once, repeated 3 times), was recognized as the candidate inhibitory peptides, and those with 0.00 or 0.50 were chosen as the controls (Table 4). Based on the existing computing equipment, the screening process consumed 903.40 s in total (0.08 s/peptide). In order to verify the feasibility of our prediction model, the peptide–protein docking was conducted on the candidate ACE-inhibitory peptides. The docking results (represented as relative free energy by the platform) and some visual 3D structural diagrams are displayed in Table 4 and Figure 6. There was a significant difference of the relative free energy between the candidate inhibitory peptides and the controls (both *p* < 0.05), indicating that the affinity between the candidate inhibitory peptides and ACE enzyme was evidently greater. It is worth noting that the speed of the working platform was largely affected by the length of the peptide participating in the docking task, and its docking speed is 480.00 up to ~1680.00 s/peptide, which was much slower than our XGBoost model. 

## 4. Discussion

In this study, three benchmark datasets (ACEIP214, ACEIP1378, and ACEIP3306) of ACE-inhibitory peptides with the same numbers of positive and negative samples were established from three public databases. The results showed that ACEIP3306 performed the best in the 5-fold cross-validation process regardless of the algorithms we chose, followed by ACEIP1378 and finally ACEIP214, suggesting that the size of the training dataset might affect the abilities of the machine learning; that is, increasing the size of the dataset may enable the model to exert the deeper features, thus improving its prediction accuracy [37]. For the test between different datasets and that of the total dataset, the AUC values of the two supplementary experiments (Supplementary Materials) were slightly lower than that of the single dataset, which indicated that the replacement of the test data will cause a certain disturbance to the model. The possible reason is that slight differences among the different databases have existed in the standard to prove whether a given peptide is antihypertensive or not, which may influence the cognitive ability of the models. As a result, unifying and perfecting the specific standards and protocols would be beneficial to improve the generalizations of the models. However, it is undeniable that its AUC was still relatively excellent, with strong generalization performance. 

With the developments of artificial intelligence technology, machine learning has been applied in the drug screening of active substance. In the research of ACE inhibitors screening, Ya et al. [10] used the SVM algorithm and ligand-based QSAR model to predict ACE inhibitors, resulting in an excellent accuracy. Guan et al. [11] successfully established the QSAR model using orthogonal signal correction combined with SVM (OSC-SVM) through 268 peptides, which showed a relatively excellent fitting accuracy and generalization ability in the task of predicting ACE inhibitory peptides. In the screening tasks of other targets, Cai et al. [12] employed machine learning models such as Plain Bayes and recursive partition algorithms to predict DPP-IV inhibitors; they established 247 sub-models based on 1307 known DPP-IV inhibitors, and the final overall prediction accuracy exceeded 80.00%. Chandra et al. [13] also designed an SVM algorithm to predict DPP-IV inhibitors with the Matthew correlation coefficient in the external test set of 0.88, and they have further applied the method to Web programs. Yi et al. [14] screened the anticancer peptide utilizing long short-term memory, which achieved a better performance than the traditional machine learning method. In the present study, the overall effect of our XGBoost model was better than that of the three dominating machine learning methods, due to the use of a superior algorithm and larger datasets.

As a novel Boosting ensemble learning algorithm, it is innovative to adopt XGBoost in the task of antihypertensive peptides, even though the algorithm has been proven to have excellent performance in other fields [38,39]. In this study, the primary structural feature of the peptides described by the PseAAC algorithm was inputted to the XGBoost model. Using 5-fold cross-validation, the Acc, Sens, Spec, Prec, and AUC were calculated as the indicators to test the model. Meanwhile, three classic machine learning methods (SVM, RF, and K-NN) were employed to screen and predict the properties of the peptides. Our results showed that the XGBoost model performed better than the other algorithms based on all the three datasets. More specifically, the regularization terms were introduced into the XGBoost model to control the complexity of the model, which not only simplified the learning model but also prevented over fitting. Besides, for the samples that occasionally miss part of the features, XGBoost can automatically learn its splitting direction. The XGBoost model also uses a greedy algorithm to enumerate all possible split points, which benefits the generating of the optimal tree structure.

The test dataset was composed of over 10,000 sequences from six key proteins in bovine milk, which were used to verify the reliability of our optimal model (training with the dataset ACEIP3306). A possibility value represented as the ACE-inhibitory degree was obtained for every sequence through the XGBoost model. The results of three parallel experiments were consistent with the peptide–protein docking results, thus proving the feasibility of the machine learning method as a novel auxiliary tool for ACE inhibitory peptide screening. It is worth noting that the screening speed of our method was remarkably faster than the traditional docking technology, indicating its potential to achieve a high-throughput and be a rapid screening tool. It should be emphasized that the short peptides cut from the six key proteins in bovine milk were employed to predict their antihypertensive properties, supporting the strategy that employs machine learning algorithms to predict the function of peptides derived from food protein with known sequences.

It is acknowledged that flaws still exist in our algorithm. Due to the lack of definitively validated non-ACE-inhibitory peptides, the negative samples in our datasets were replaced by random peptides, which were inevitably mixed with some ACE inhibitors. This possibility will doubtlessly reduce the prediction accuracy of the model. In addition, this research focuses on the establishment of theoretical methods, instead of using digestive enzyme hydrolysis, but chose the k-mer method. The k-mer sequences in our test datasets, gained from proteins in bovine milk, were obtained by theoretical segmentation algorithms without considering their biological activity and feasibility in a real situation. As a result, the feasibility of the extracted antihypertensive peptides from natural food still depends on the development and advance in enzyme cleavage technology in the future. Moreover, this research mainly focuses on the application of machine learning and has not yet been involved in in vitro experimental verification, which needs to be further improved in the future.

## 5. Conclusions

In this study, a method of utilizing PseAAC to extract the primary structural features of peptides and then establishing the XGBoost model to predict their antihypertensive properties were proposed. This method achieved excellent performance in the task of antihypertensive peptides screening, which was better than the dominating machine learning models, including the SVM, RF, and K-NN algorithms. Using the XGBoost model, the predictions of antihypertensive peptides derived from milk protein was consistent with the peptide–protein docking results, and was more efficient. The method herein can be used to discover new food-derived antihypertensive peptides. However, what cannot be ignored is that the experiment also has defects, such as a lack of consideration of the actual conditions. In the future, in vitro wet experiments will be performed for further improvement of the current results.

## Figures and Tables

**Table 1:** The information of the six key proteins in bovine milk obtained from UniProt.

Entry	Entry Name	Protein Names	Gene Names	Organism	Length
P02666	CASB_BOVIN	Beta-casein	CSN2	Bos taurus (Bovine)	224
P02668	CASK_BOVIN	Kappa-casein	CSN3 CSN10 CSNK	Bos taurus (Bovine)	190
P02662	CASA1_BOVIN	Alpha-S1-casein	CSN1S1	Bos taurus (Bovine)	214
P02663	CASA2_BOVIN	Alpha-S2-casein	CSN1S2	Bos taurus (Bovine)	222
P02754	LACB_BOVIN	Beta-lactoglobulin	LGB	Bos taurus (Bovine)	178
P00711	LALBA_BOVIN	Alpha-lactalbumin	LALBA ALACTA	Bos taurus (Bovine)	142

**Table 2:** Performance of the eXtreme Gradient Boosting (XGBoost) model on the different datasets.

Name of Dataset	Acc (%)	Sens (%)	Spec (%)	Prec (%)
ACEIP214	77.49 ± 4.87	77.87 ± 4.20	77.00 ± 7.12	79.62 ± 5.72
ACEIP1378	85.47 ± 2.37	85.35 ± 2.83	85.60 ± 3.42	86.46 ± 2.78
ACEIP3306	86.50 ± 1.24	86.08 ± 1.24	86.92 ± 2.12	86.85 ± 1.87

Notes: The above results show the average value of each indicator and the corresponding 95% confidence interval (CI). Additionally, Acc, Sens, Spec, and Prec represent accuracy, sensitivity, specificity, and precision, respectively.

**Table 3:** Performance of the XGBoost model in the above experiments.

Datasets for Training	Datasets for Testing	AUC (%)
ACEIP3306	ACEIP214	88.18 ± 1.27
ACEIP3306	ACEIP1378	86.33 ± 0.98
80% of total dataset	The rest of total dataset	91.85 ± 0.82

**Table 4:** Results of the screening and the corresponding docking scores (relative free energy).

Prediction Probability of Positive Samples	Peptide Sequence	The Docking Scores
0.99+ (candidate inhibitors)	RLNFLKKI	−212.37
	SITRINKKI	−205.44
	ILTCLV	−174.48
	LVVTILA	−172.51
	DQVKRNA	−172.46
	LTCL	−138.38
	ILTC	−138.35
	LILT	−136.18
0.50	PQN	−129.85
	ACLV	−115.72
	ESLS	−113.95
	LKK	−111.86
	LEI	−100.82
	LQD	−100.31
	QLE	−96.19
	EIV	−87.36
0.00	EDV	−86.31
	KEK	−84.55
	KV	−83.00
	KED	−80.58
	GKE	−78.43
	SEE	−62.48
	EDS	−60.17
	DE	−48.25

## Data Availability

The data presented in this study are available on request from the corresponding author.

## Supplementary Materials

The following are available online at https://www.mdpi.com/2304-8158/10/3/550/s1.
