# Supplementary figures and images for: A Novel Machine Learning Strategy for the Prediction of Antihypertensive Peptides Derived from Food with High Efficiency
Source: Foods. 2021 Mar 6;10(3):550. doi: 10.3390/foods10030550 (PMC7999667; doi:10.3390/foods10030550)

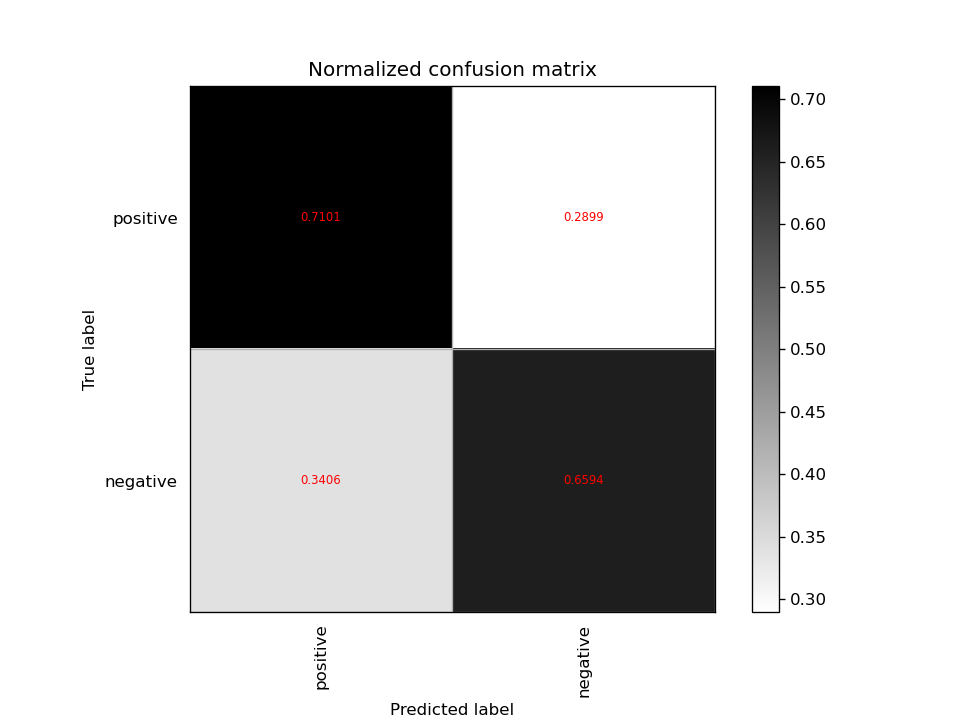

Supplement: Supplementary file 1 [file foods-10-00550-s001.zip › attachment/Confusion matrix-KNN/1378-1.png]

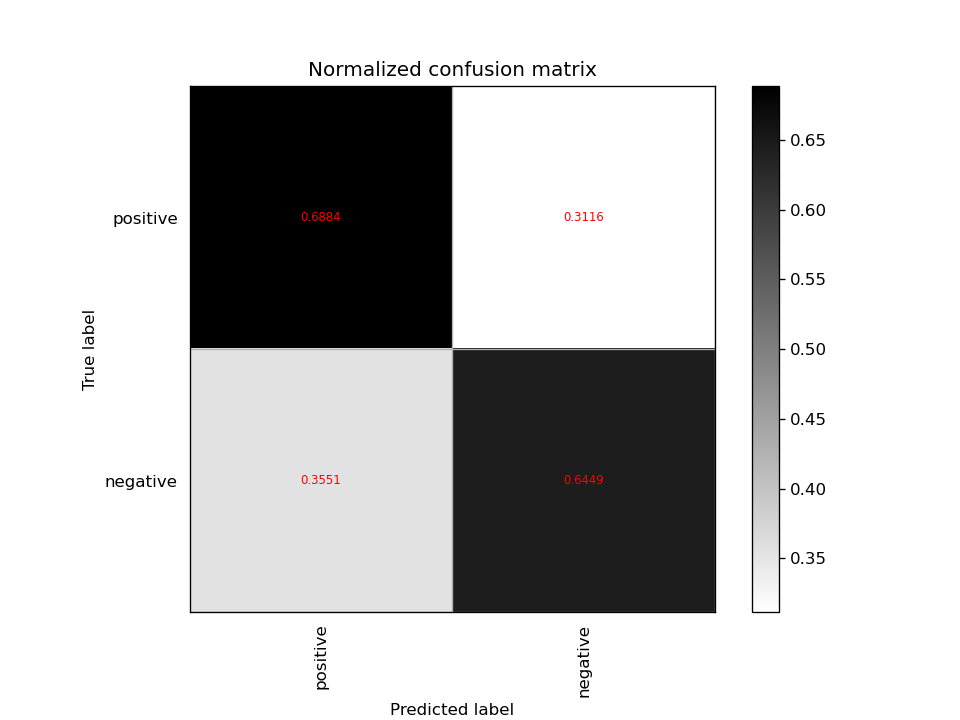

Supplement: Supplementary file 1 [file foods-10-00550-s001.zip › attachment/Confusion matrix-KNN/1378-2.png]

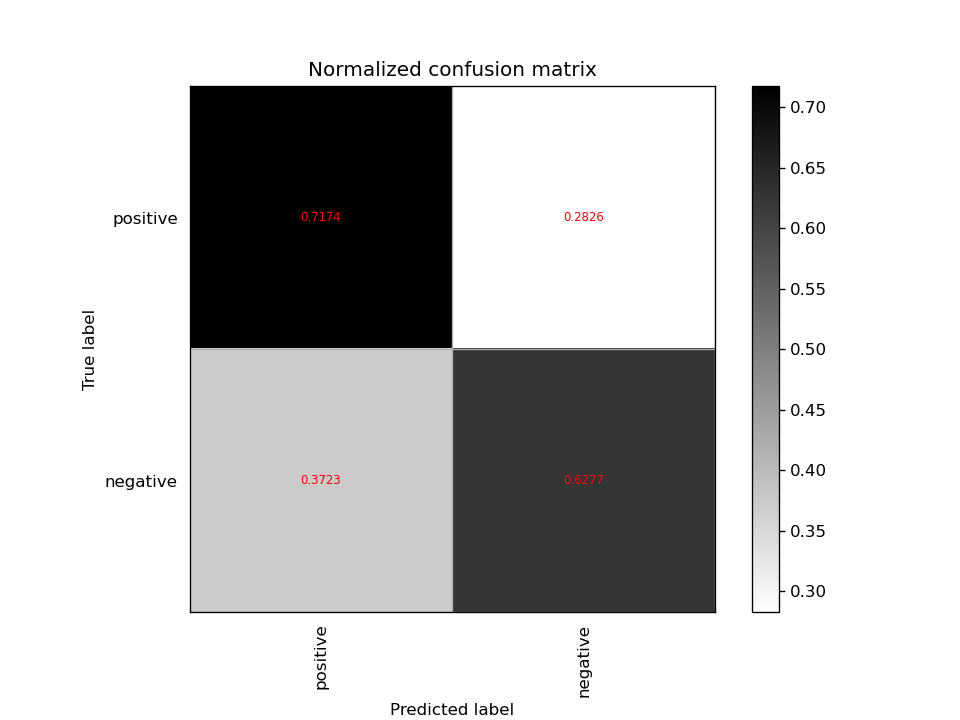

Supplement: Supplementary file 1 [file foods-10-00550-s001.zip › attachment/Confusion matrix-KNN/1378-3.png]

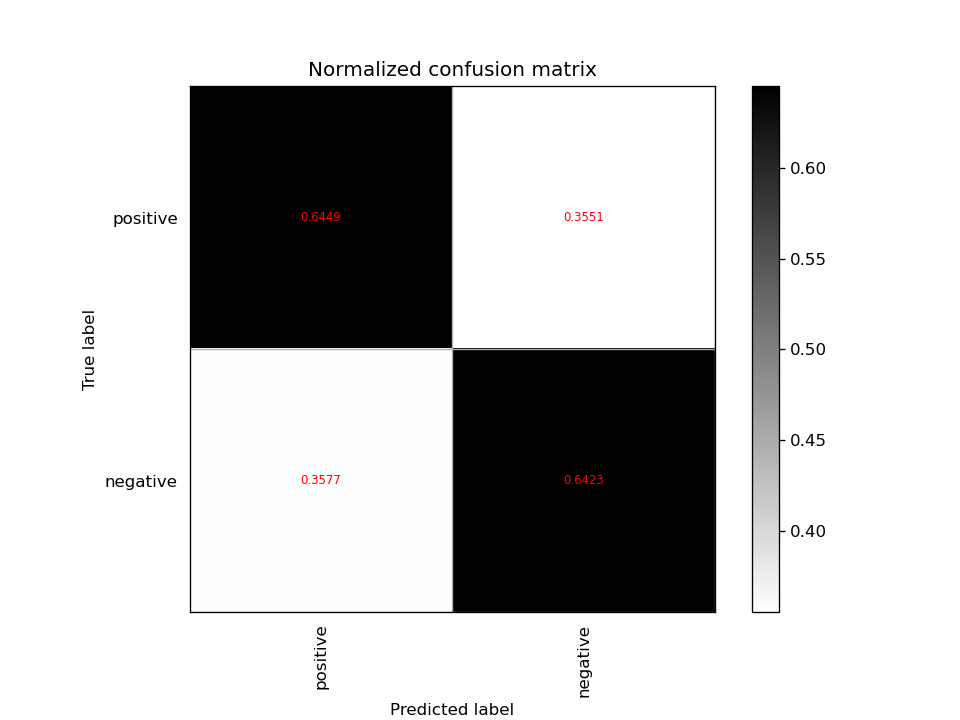

Supplement: Supplementary file 1 [file foods-10-00550-s001.zip › attachment/Confusion matrix-KNN/1378-4.png]

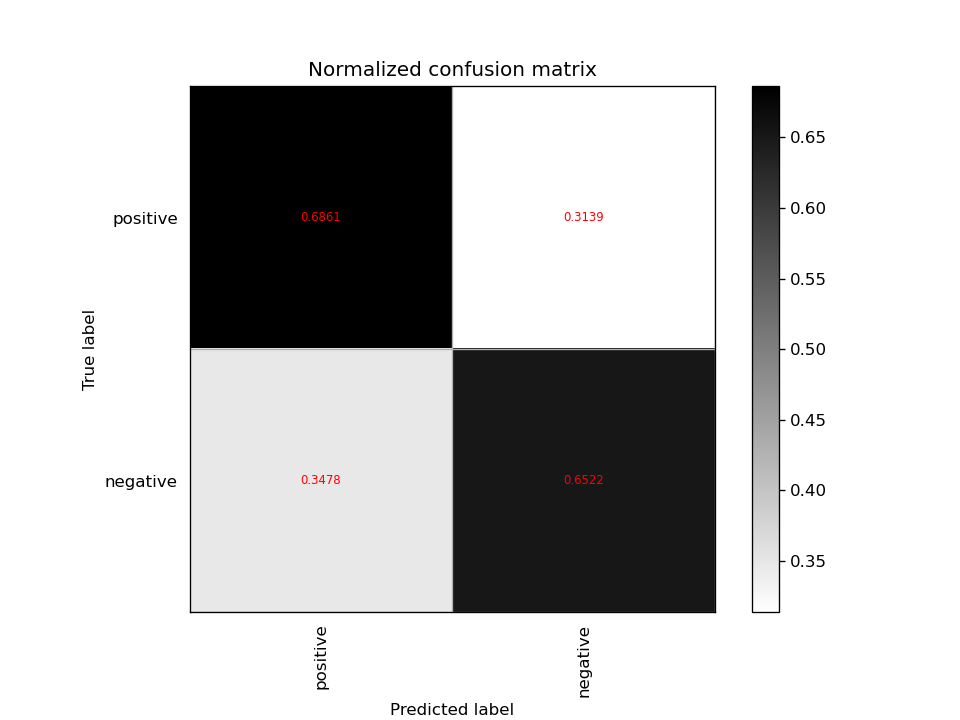

Supplement: Supplementary file 1 [file foods-10-00550-s001.zip › attachment/Confusion matrix-KNN/1378-5.png]

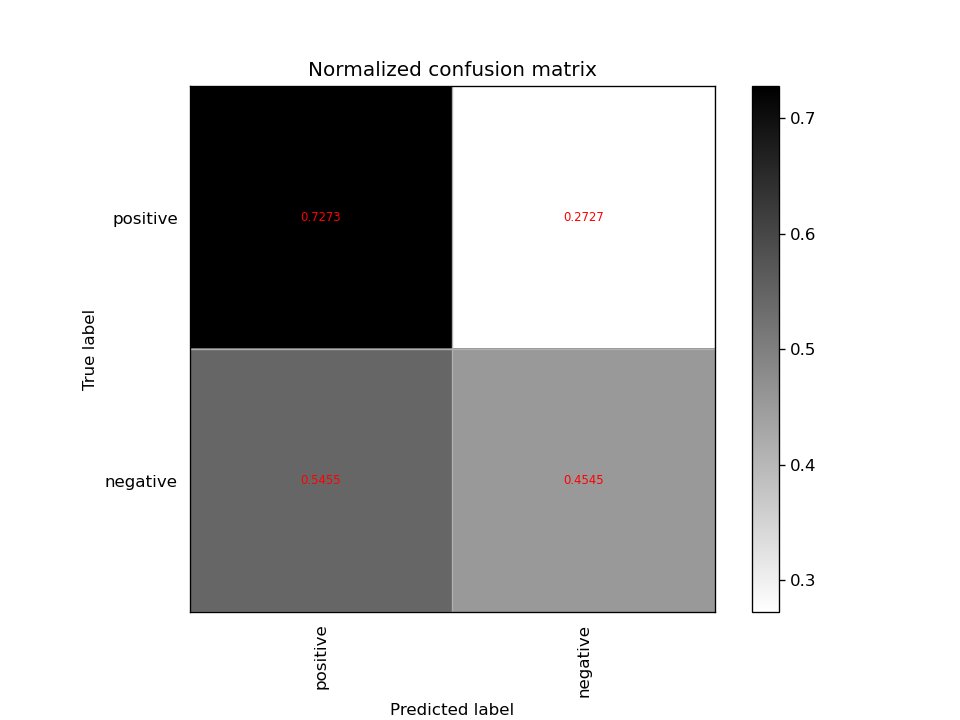

Supplement: Supplementary file 1 [file foods-10-00550-s001.zip › attachment/Confusion matrix-KNN/214-1.png]

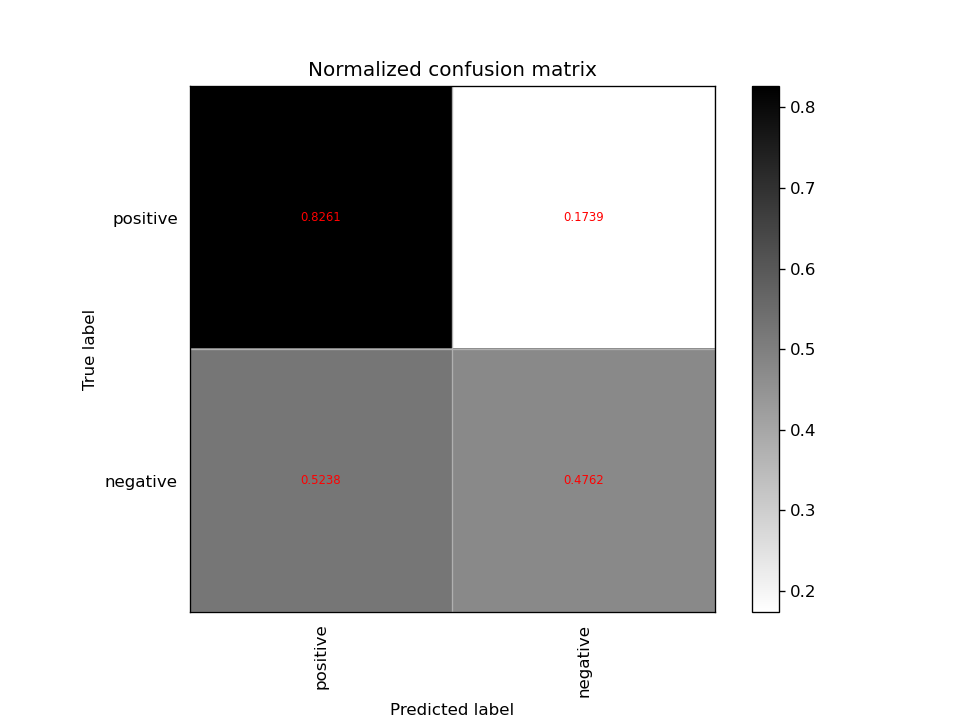

Supplement: Supplementary file 1 [file foods-10-00550-s001.zip › attachment/Confusion matrix-KNN/214-2.png]

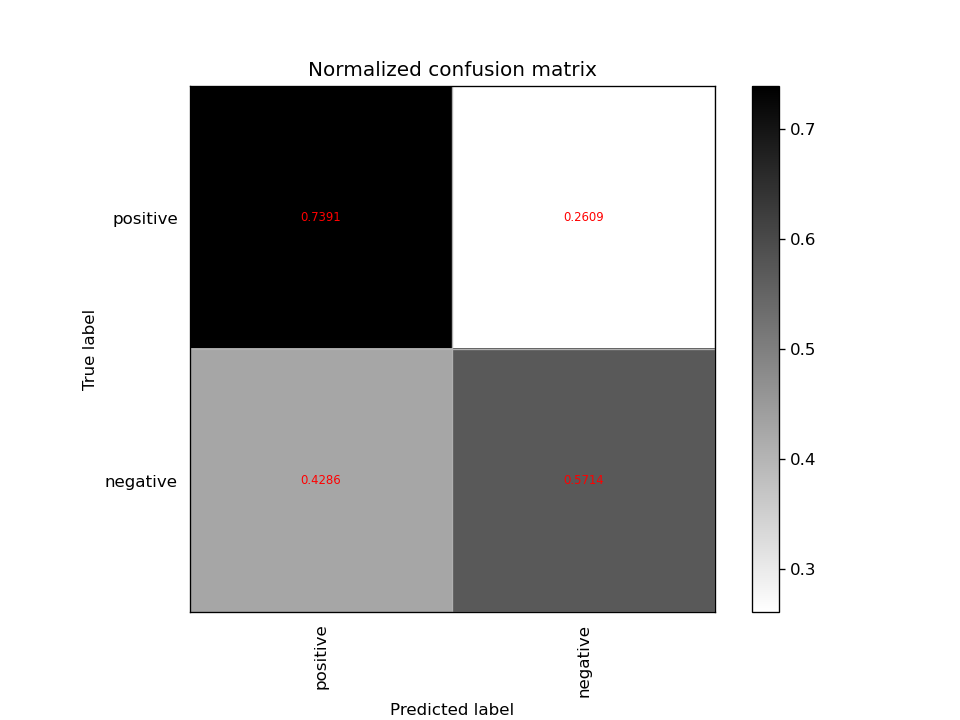

Supplement: Supplementary file 1 [file foods-10-00550-s001.zip › attachment/Confusion matrix-KNN/214-3.png]

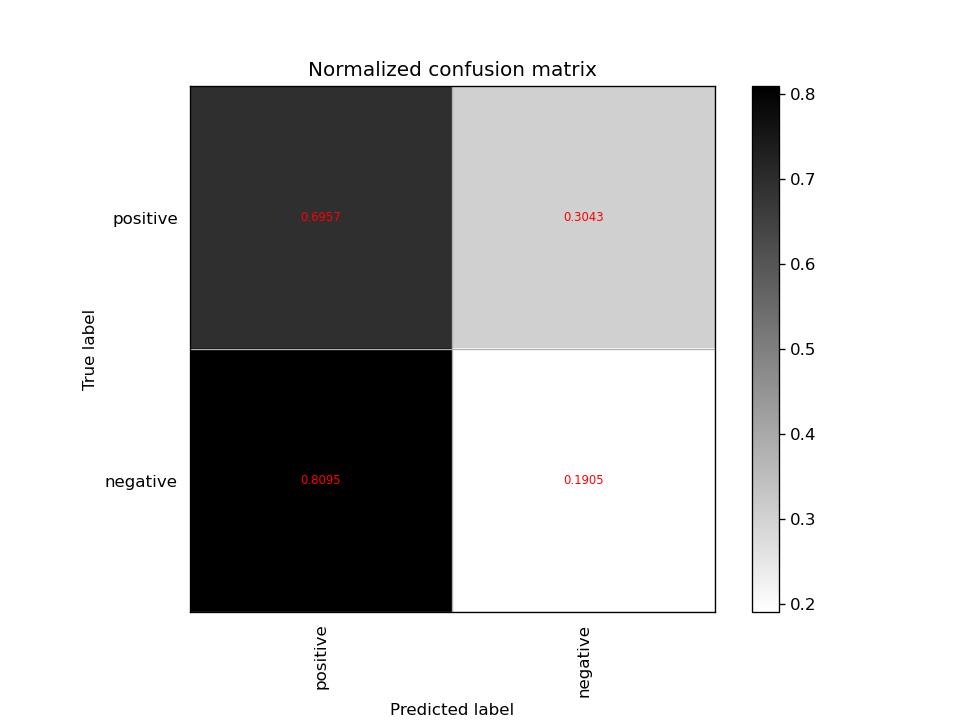

Supplement: Supplementary file 1 [file foods-10-00550-s001.zip › attachment/Confusion matrix-KNN/214-4.png]

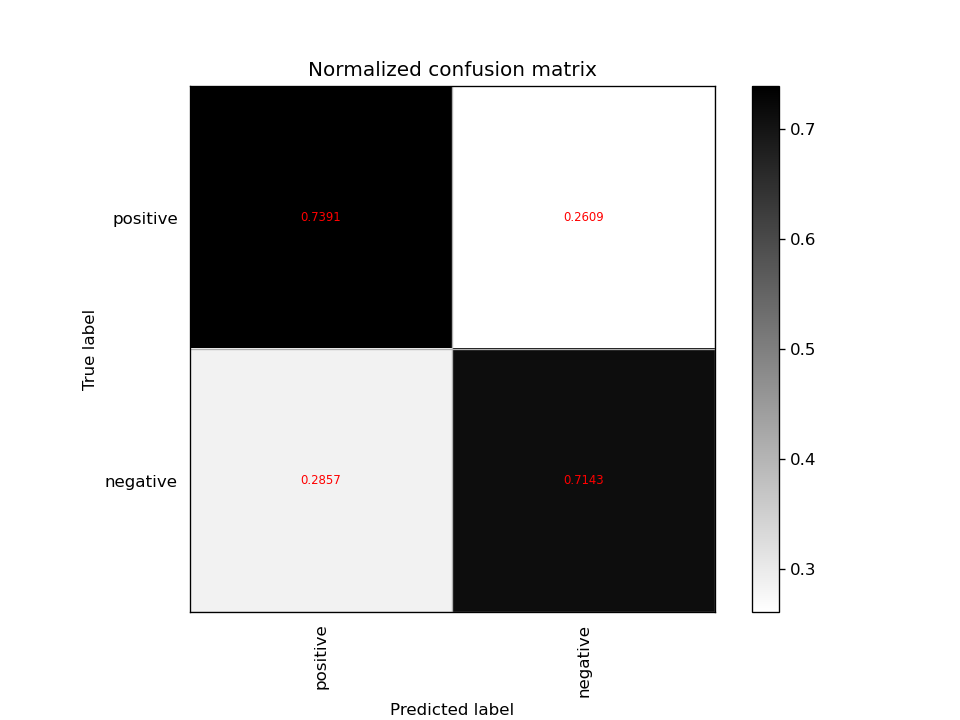

Supplement: Supplementary file 1 [file foods-10-00550-s001.zip › attachment/Confusion matrix-KNN/214-5.png]

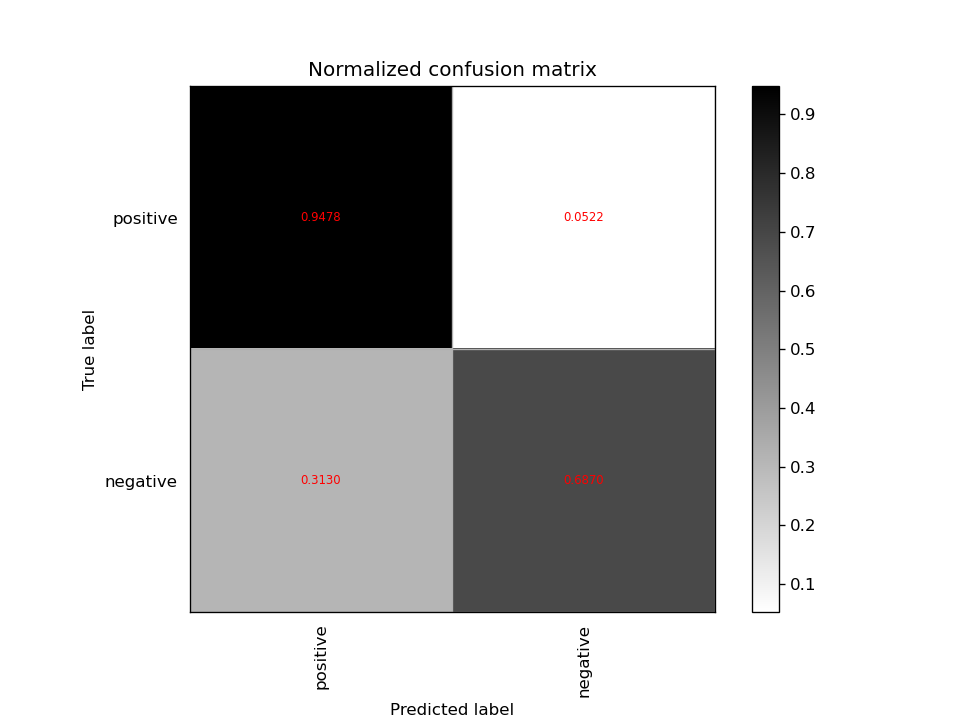

Supplement: Supplementary file 1 [file foods-10-00550-s001.zip › attachment/Confusion matrix-KNN/3306-1.png]

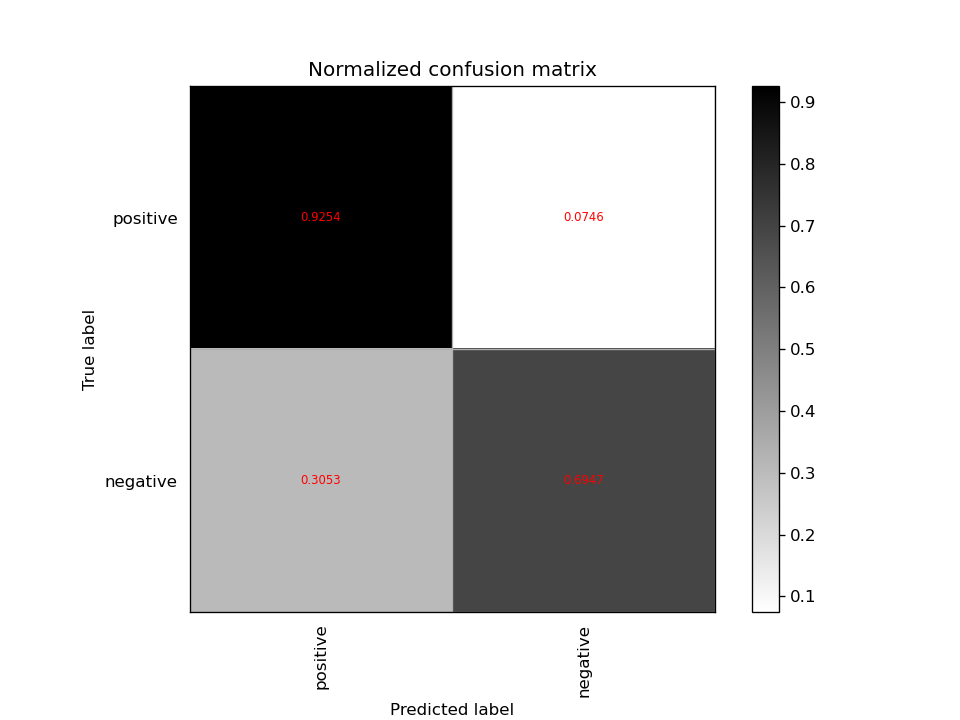

Supplement: Supplementary file 1 [file foods-10-00550-s001.zip › attachment/Confusion matrix-KNN/3306-2.png]

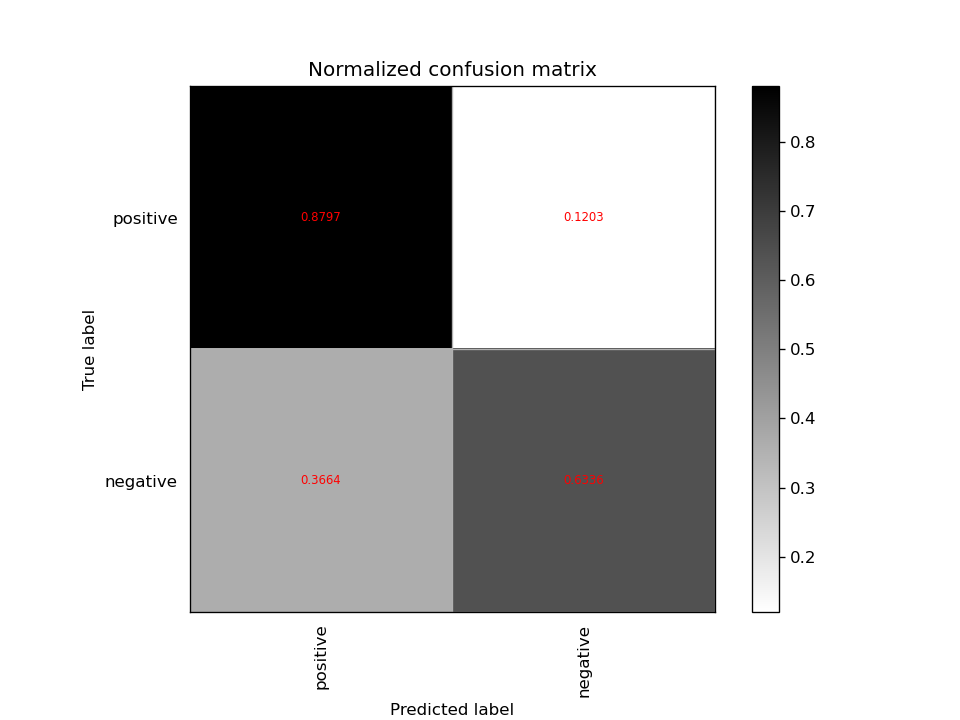

Supplement: Supplementary file 1 [file foods-10-00550-s001.zip › attachment/Confusion matrix-KNN/3306-3.png]

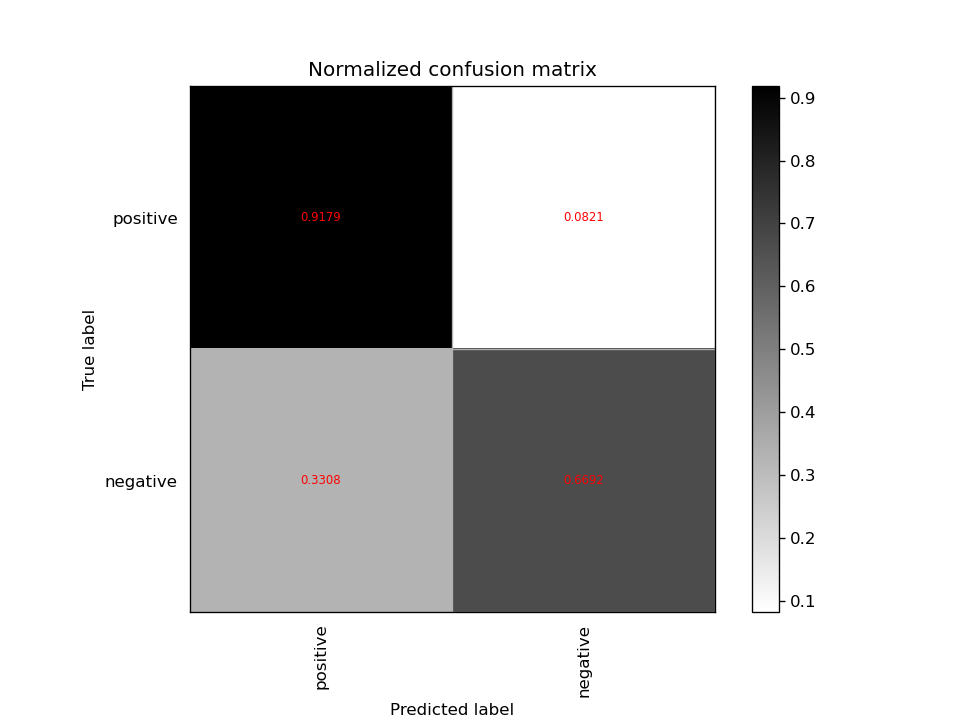

Supplement: Supplementary file 1 [file foods-10-00550-s001.zip › attachment/Confusion matrix-KNN/3306-4.png]

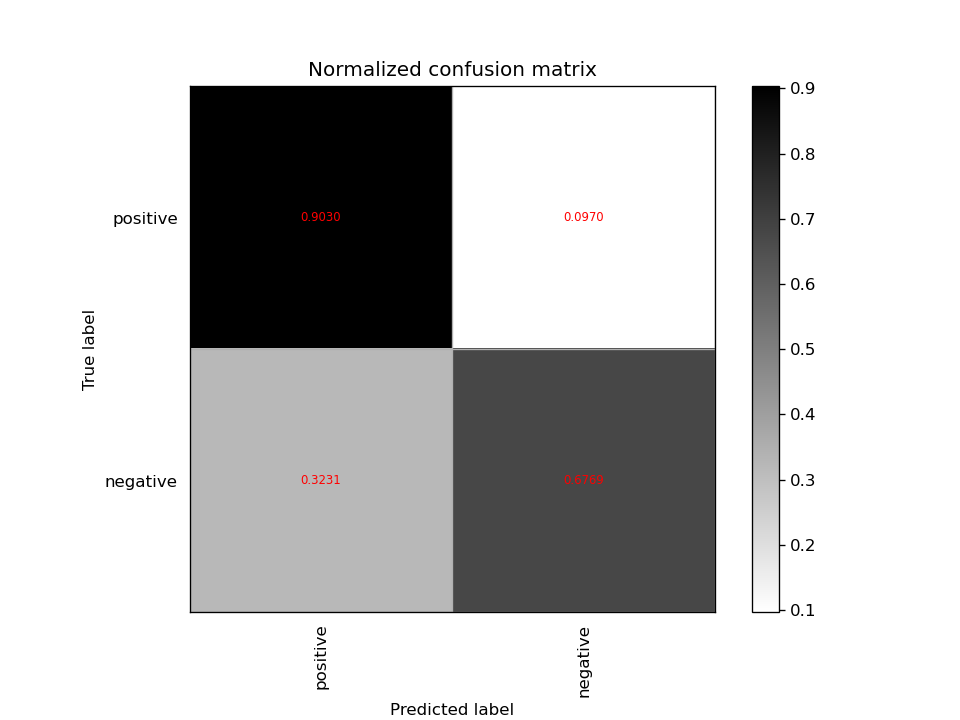

Supplement: Supplementary file 1 [file foods-10-00550-s001.zip › attachment/Confusion matrix-KNN/3306-5.png]

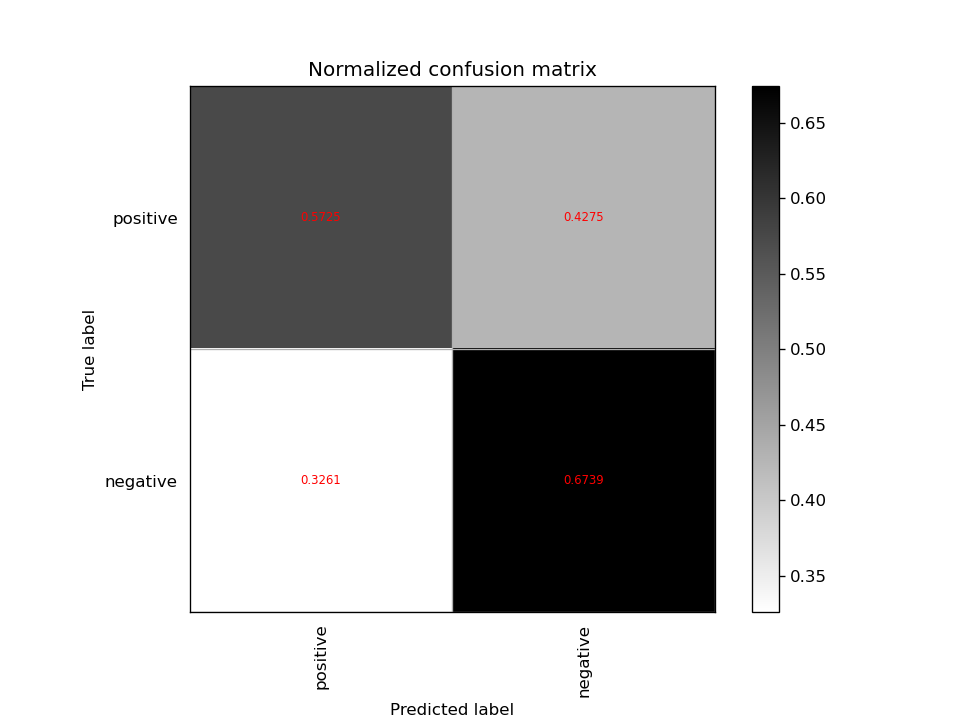

Supplement: Supplementary file 1 [file foods-10-00550-s001.zip › attachment/Confusion matrix-RF/1378-1.png]

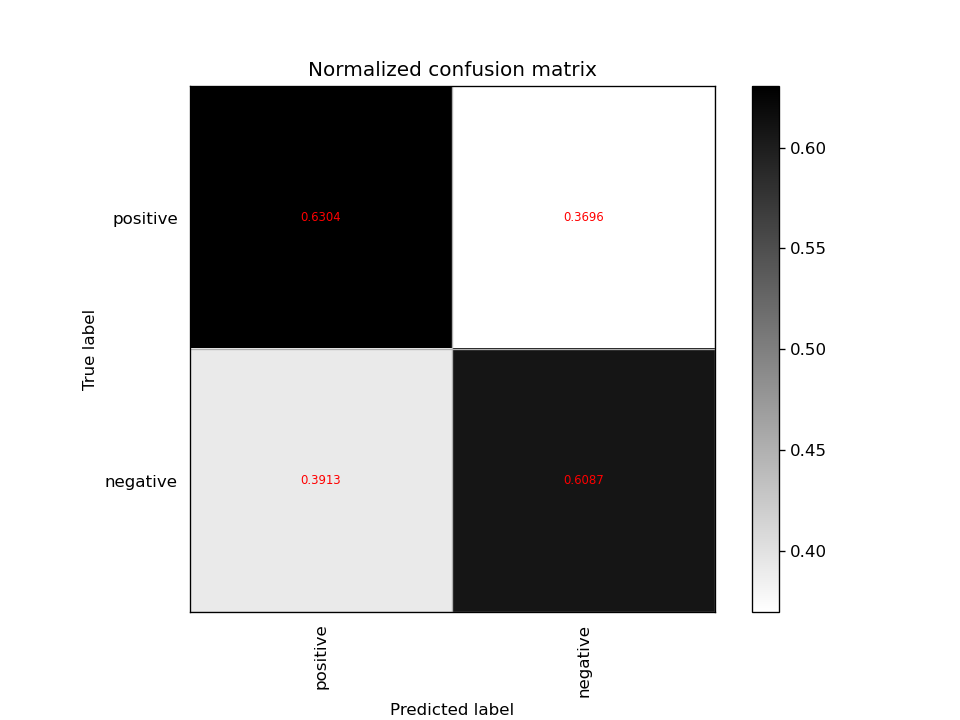

Supplement: Supplementary file 1 [file foods-10-00550-s001.zip › attachment/Confusion matrix-RF/1378-2.png]

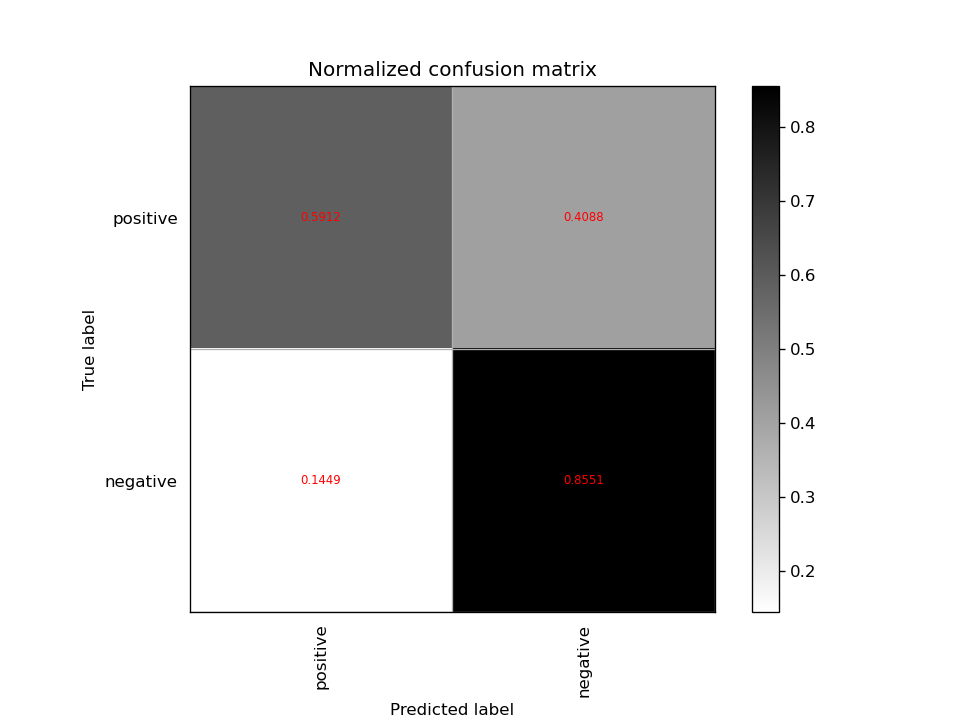

Supplement: Supplementary file 1 [file foods-10-00550-s001.zip › attachment/Confusion matrix-RF/1378-3.png]

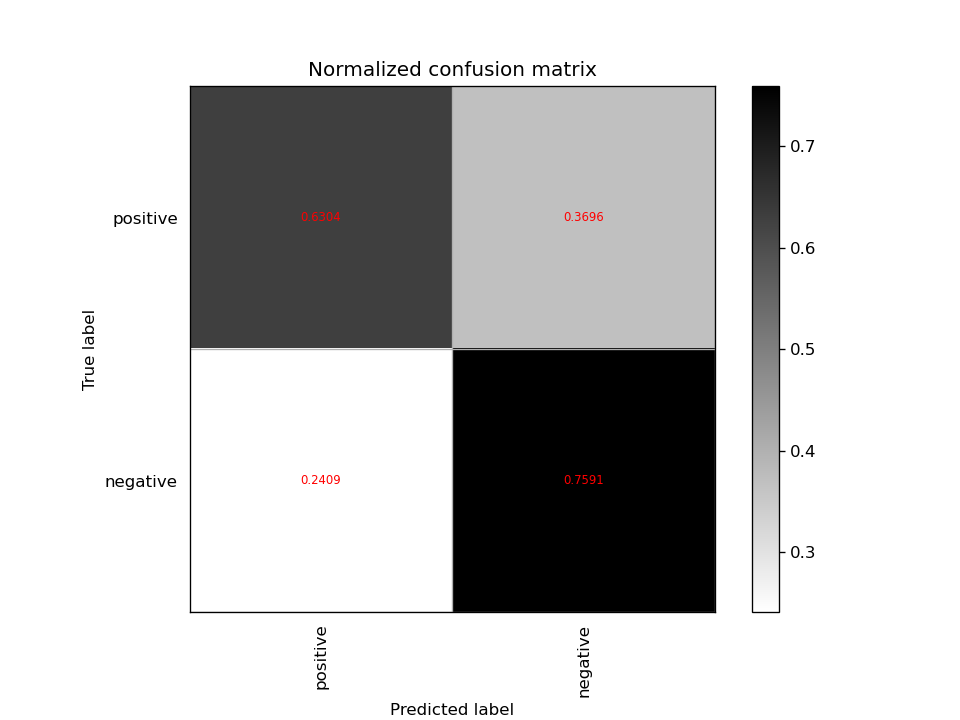

Supplement: Supplementary file 1 [file foods-10-00550-s001.zip › attachment/Confusion matrix-RF/1378-4.png]

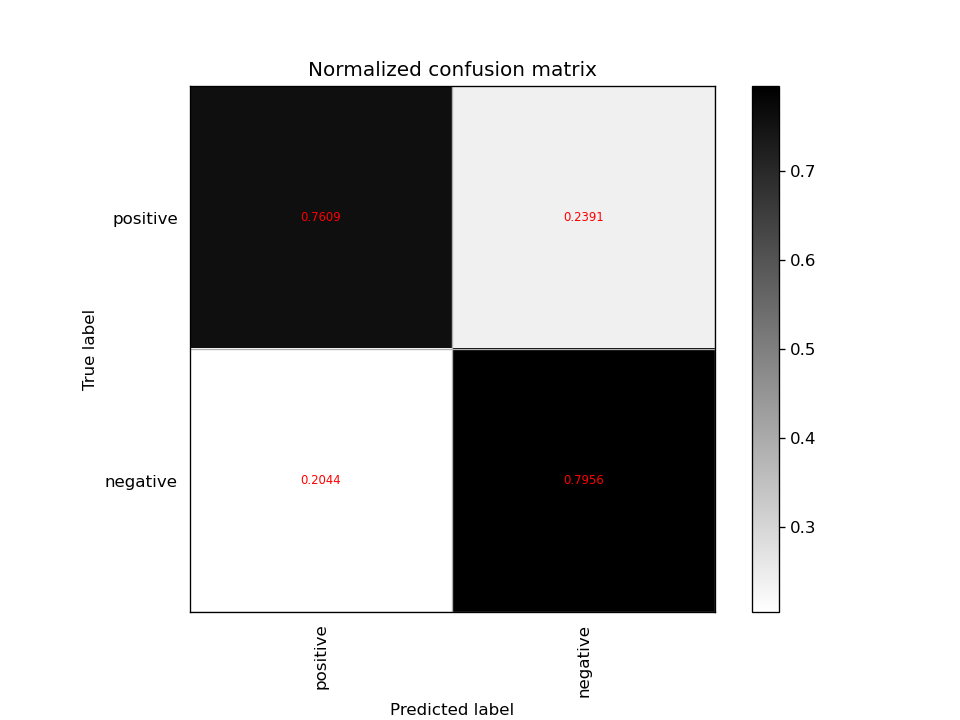

Supplement: Supplementary file 1 [file foods-10-00550-s001.zip › attachment/Confusion matrix-RF/1378-5.png]

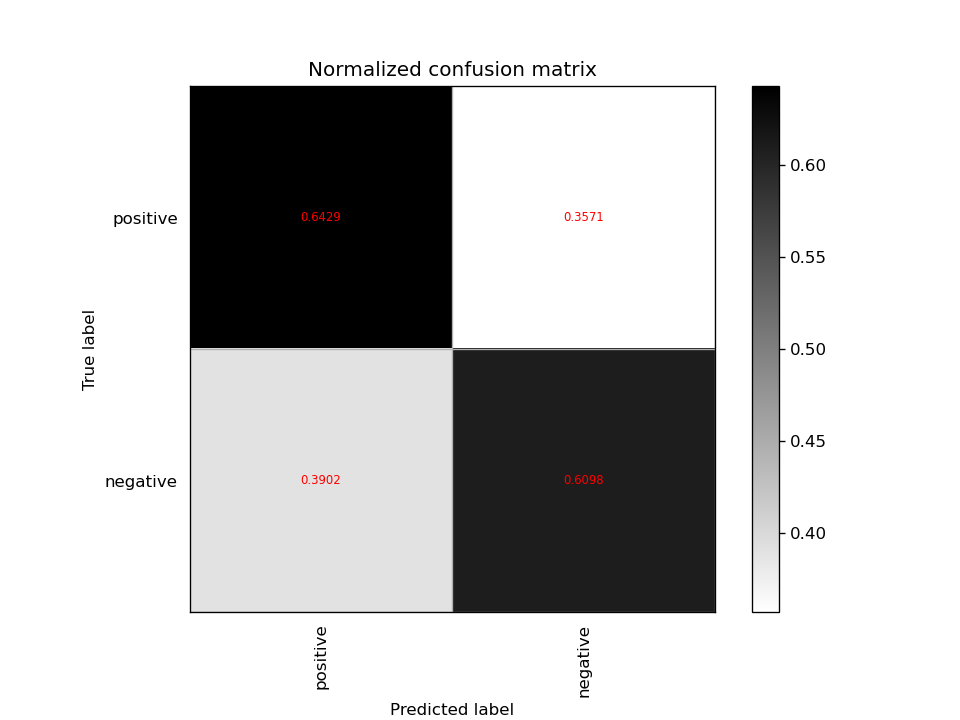

Supplement: Supplementary file 1 [file foods-10-00550-s001.zip › attachment/Confusion matrix-RF/214-1.png]

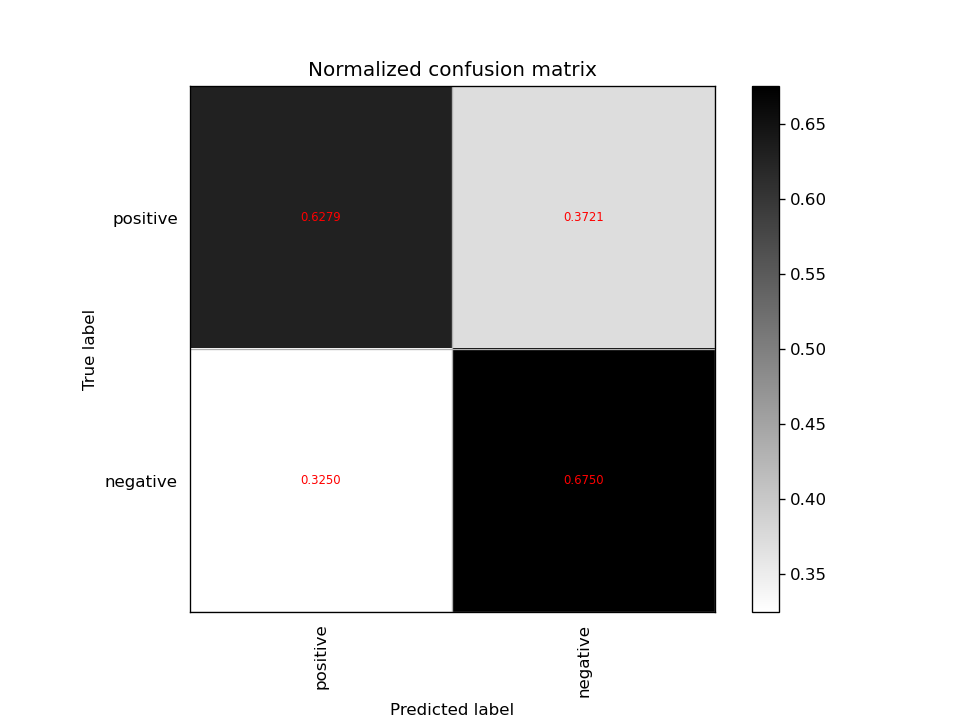

Supplement: Supplementary file 1 [file foods-10-00550-s001.zip › attachment/Confusion matrix-RF/214-2.png]

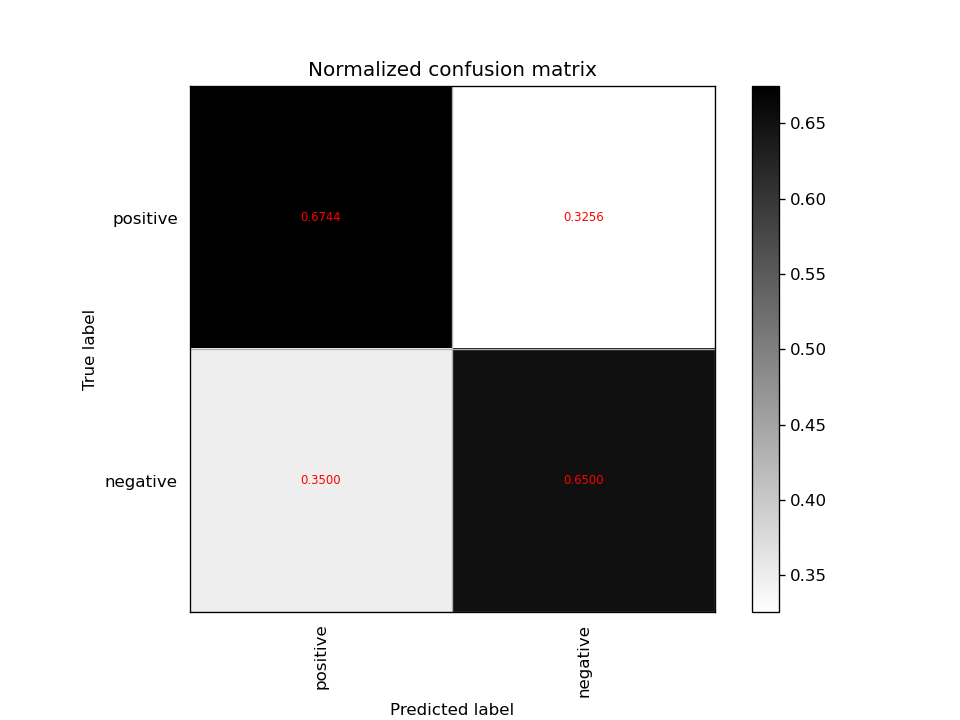

Supplement: Supplementary file 1 [file foods-10-00550-s001.zip › attachment/Confusion matrix-RF/214-3.png]

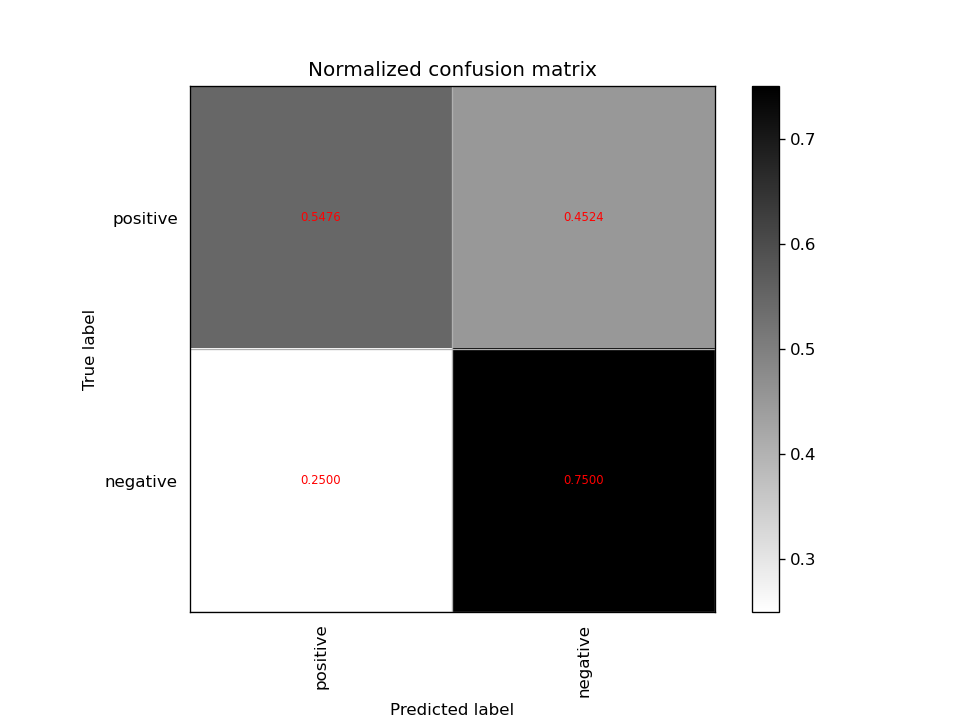

Supplement: Supplementary file 1 [file foods-10-00550-s001.zip › attachment/Confusion matrix-RF/214-4.png]

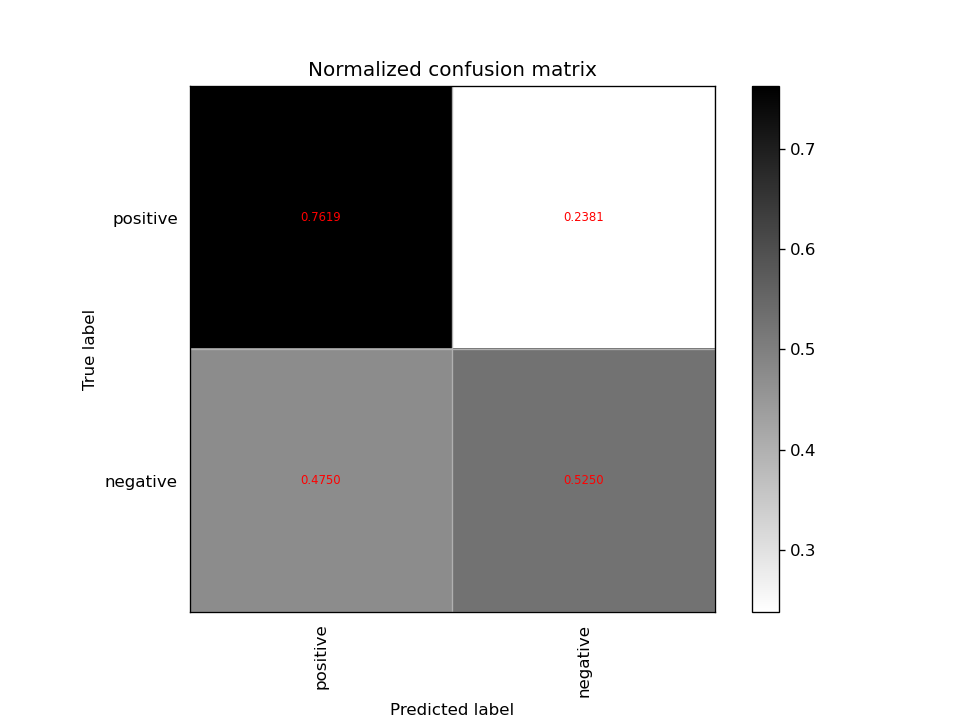

Supplement: Supplementary file 1 [file foods-10-00550-s001.zip › attachment/Confusion matrix-RF/214-5.png]

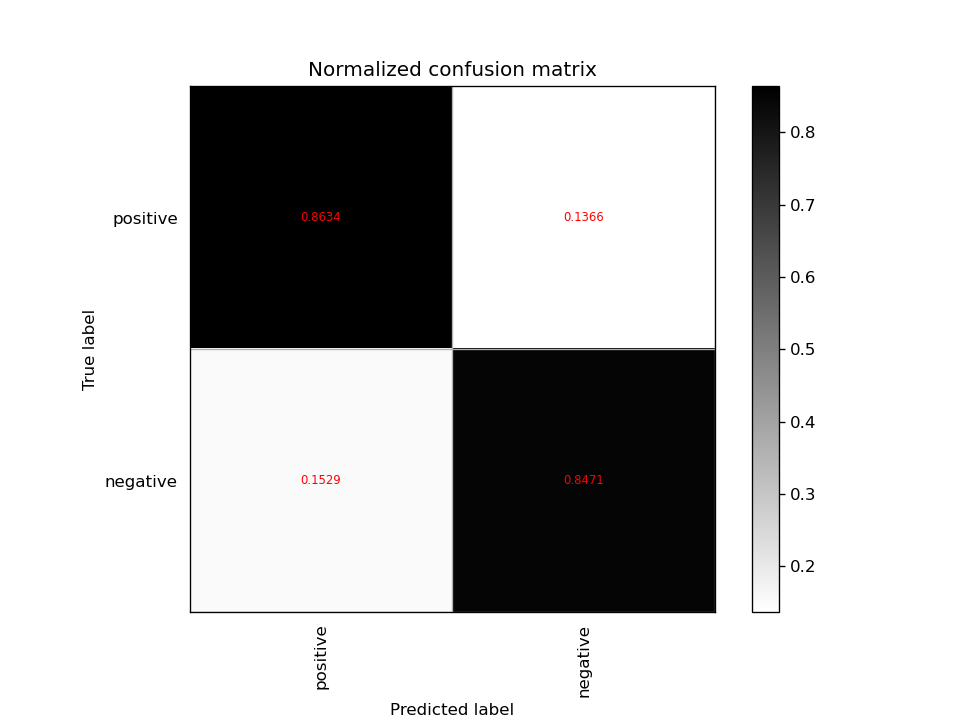

Supplement: Supplementary file 1 [file foods-10-00550-s001.zip › attachment/Confusion matrix-RF/3306-1.png]

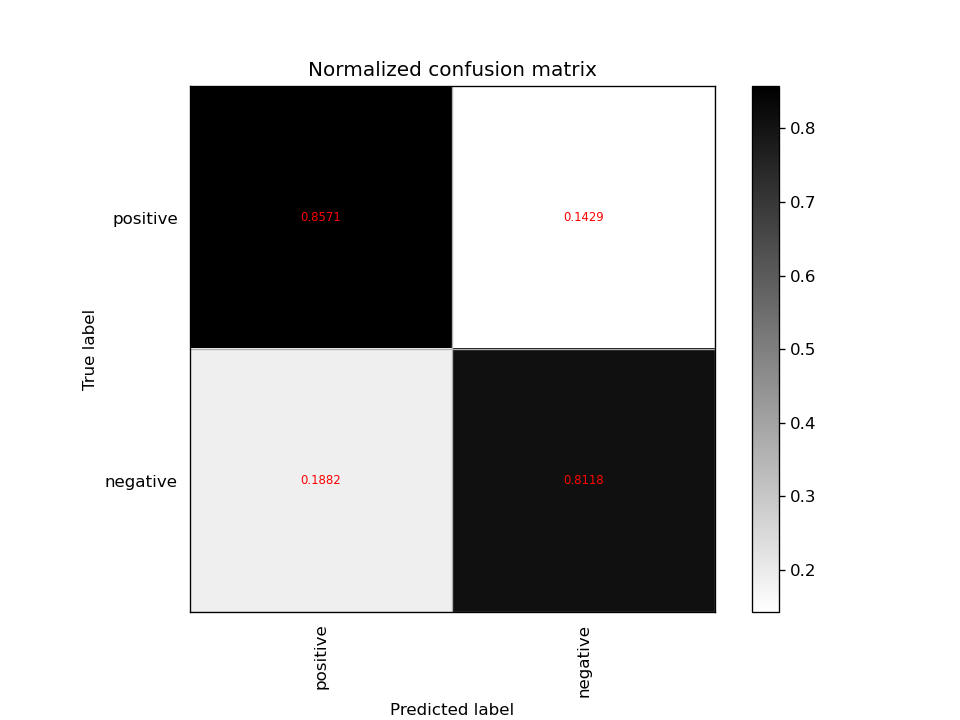

Supplement: Supplementary file 1 [file foods-10-00550-s001.zip › attachment/Confusion matrix-RF/3306-2.png]

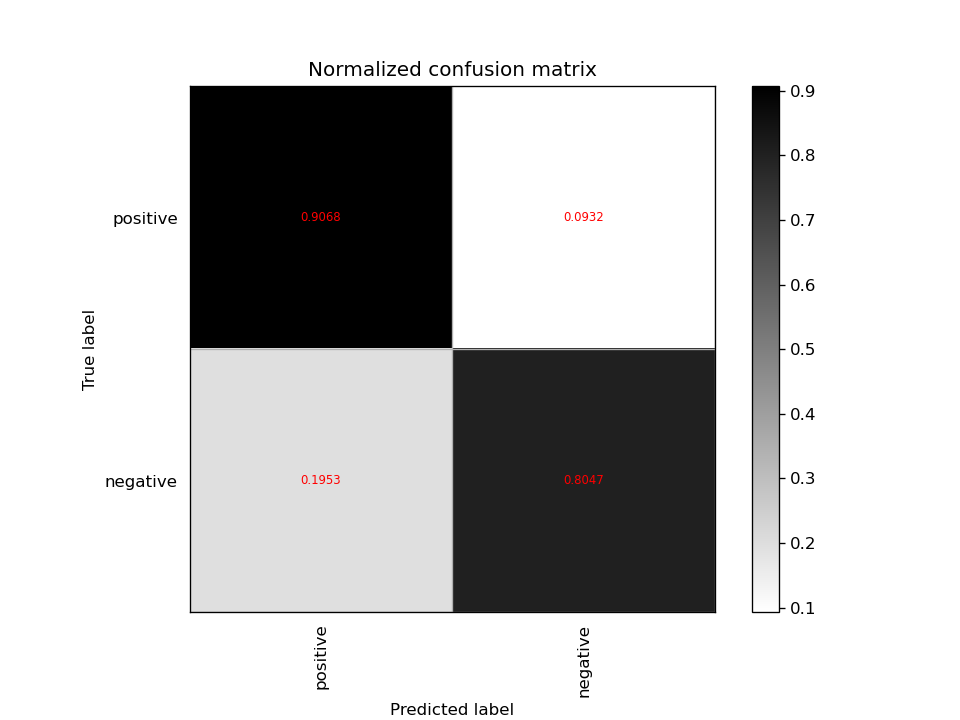

Supplement: Supplementary file 1 [file foods-10-00550-s001.zip › attachment/Confusion matrix-RF/3306-3.png]

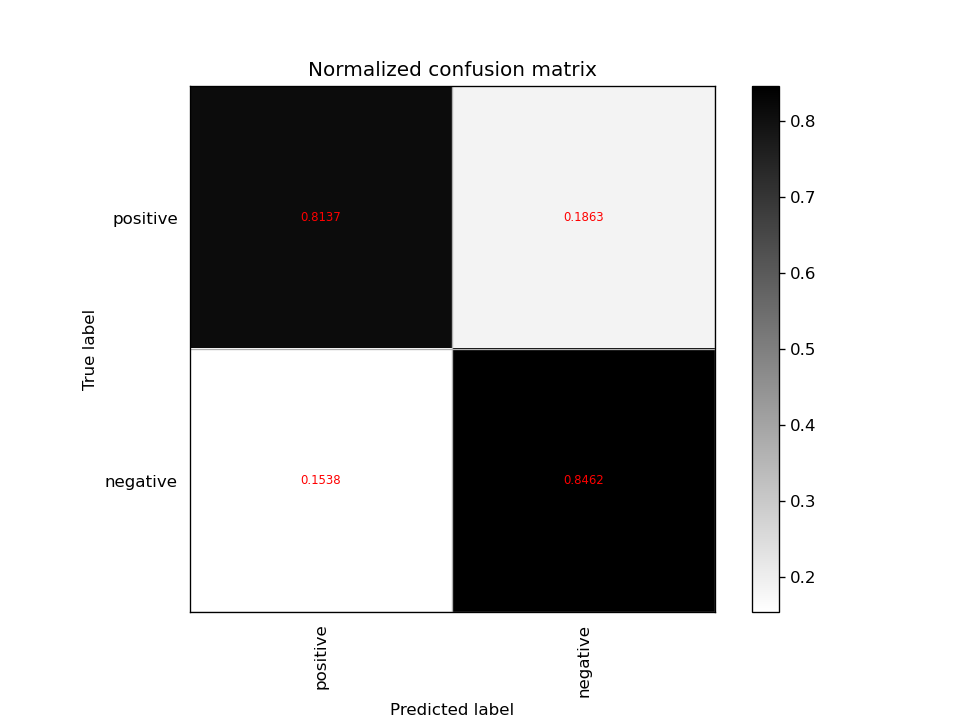

Supplement: Supplementary file 1 [file foods-10-00550-s001.zip › attachment/Confusion matrix-RF/3306-4.png]

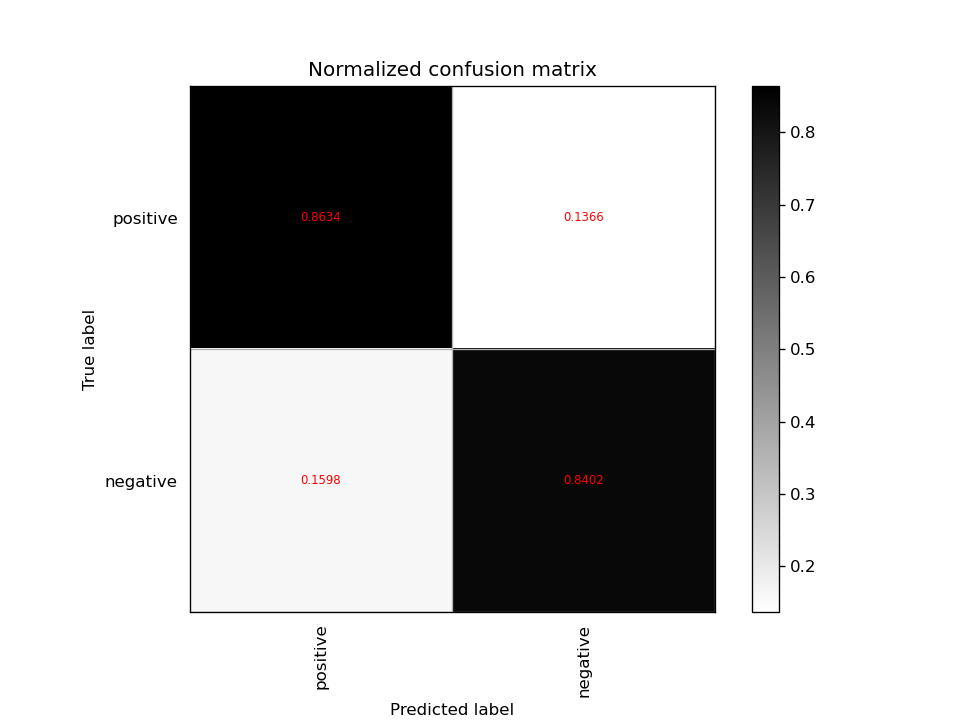

Supplement: Supplementary file 1 [file foods-10-00550-s001.zip › attachment/Confusion matrix-RF/3306-5.png]

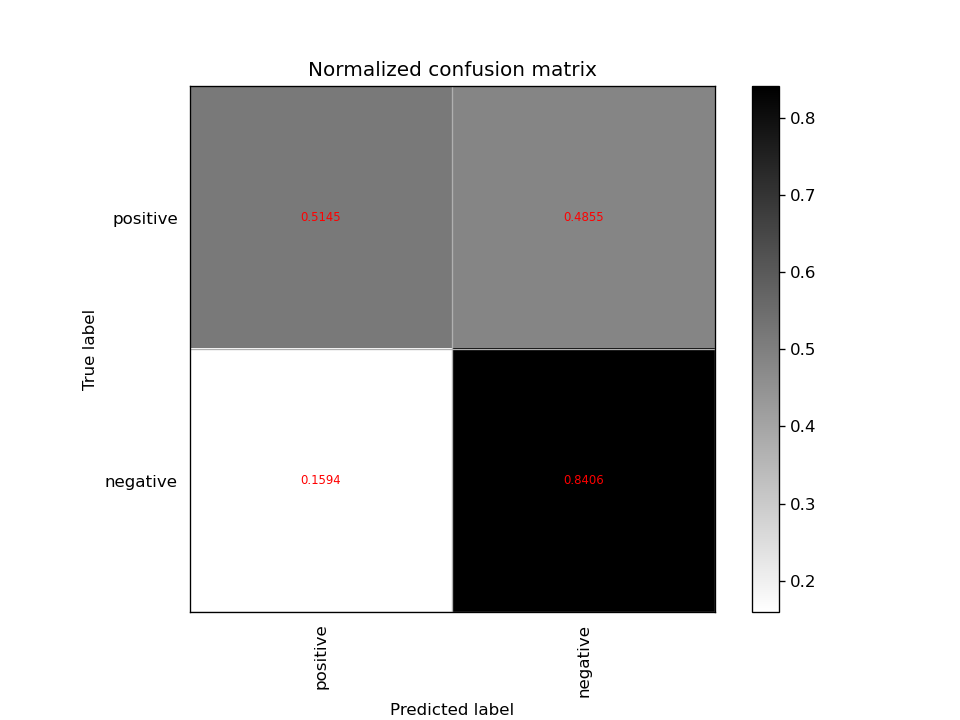

Supplement: Supplementary file 1 [file foods-10-00550-s001.zip › attachment/confusion matrix-SVM/1378-1.png]

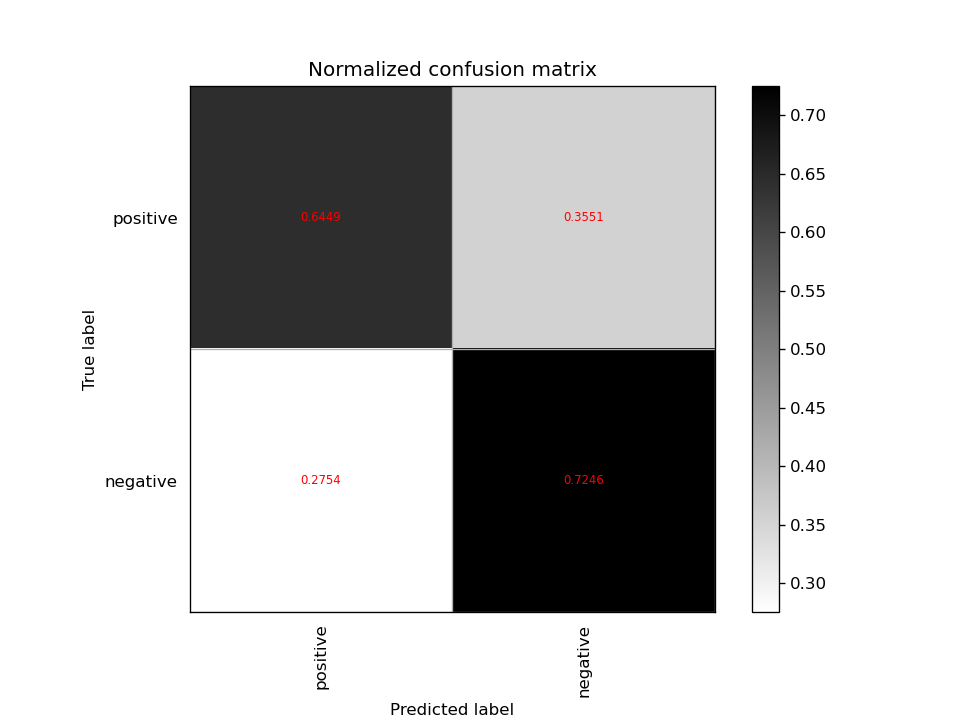

Supplement: Supplementary file 1 [file foods-10-00550-s001.zip › attachment/confusion matrix-SVM/1378-2.png]

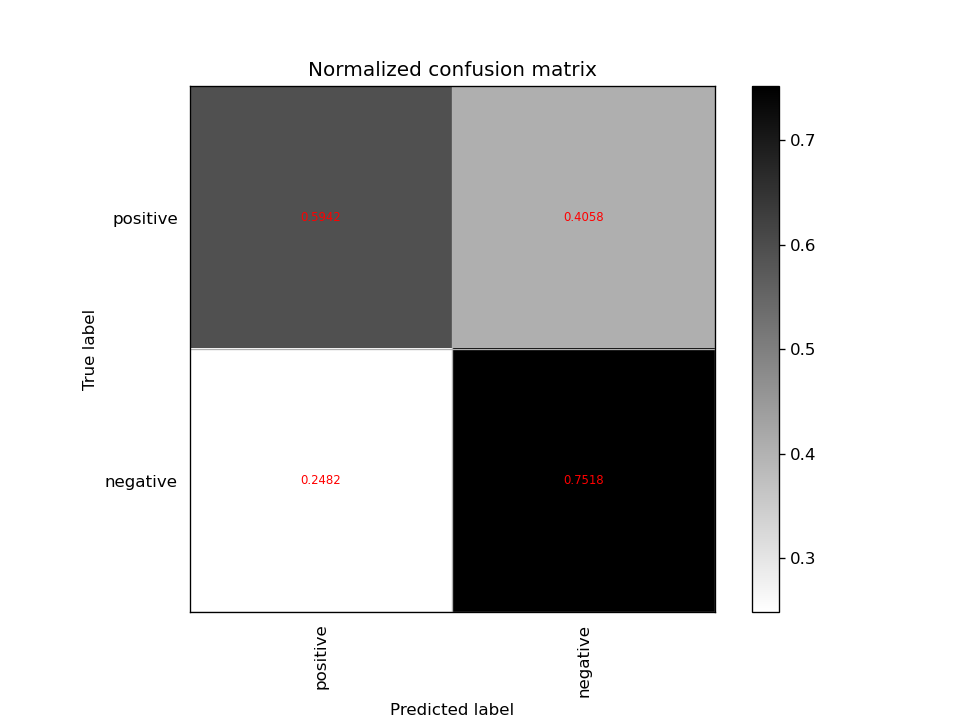

Supplement: Supplementary file 1 [file foods-10-00550-s001.zip › attachment/confusion matrix-SVM/1378-3.png]

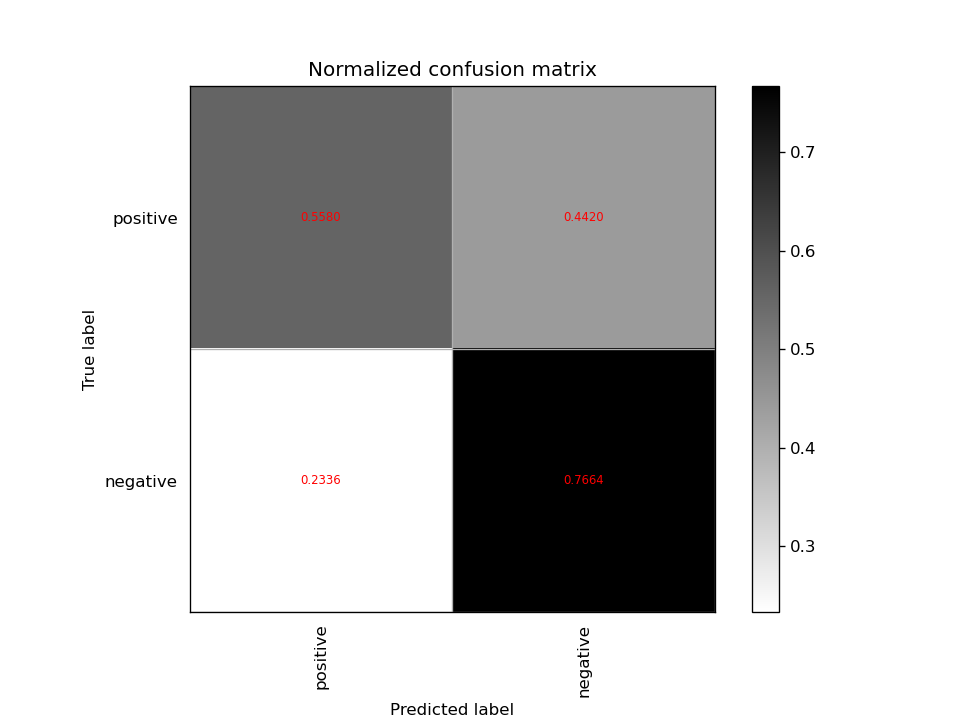

Supplement: Supplementary file 1 [file foods-10-00550-s001.zip › attachment/confusion matrix-SVM/1378-4.png]

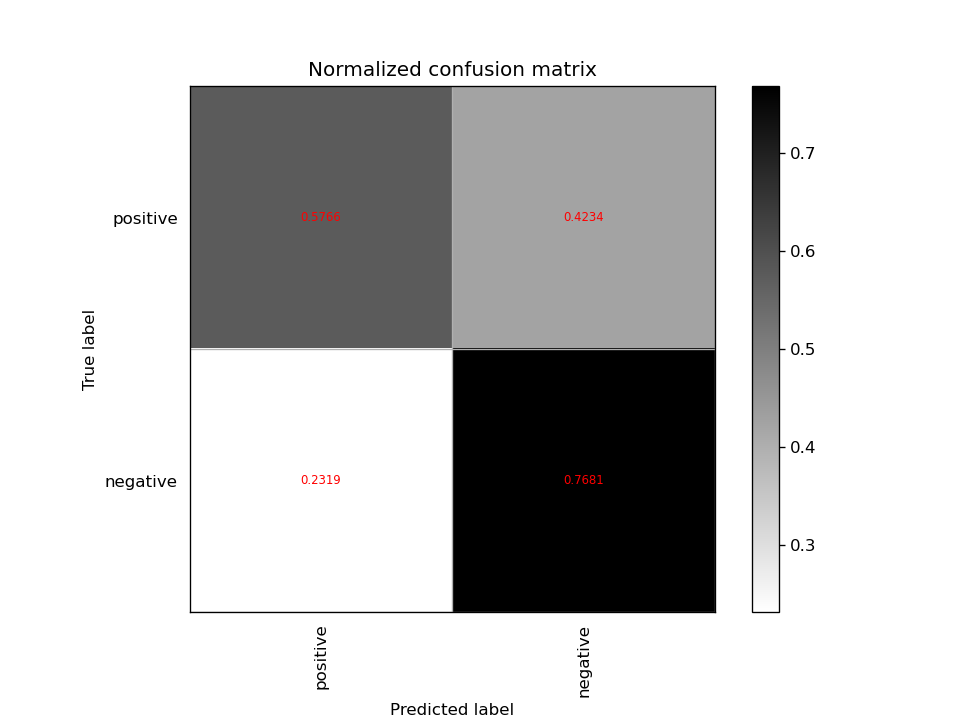

Supplement: Supplementary file 1 [file foods-10-00550-s001.zip › attachment/confusion matrix-SVM/1378-5.png]

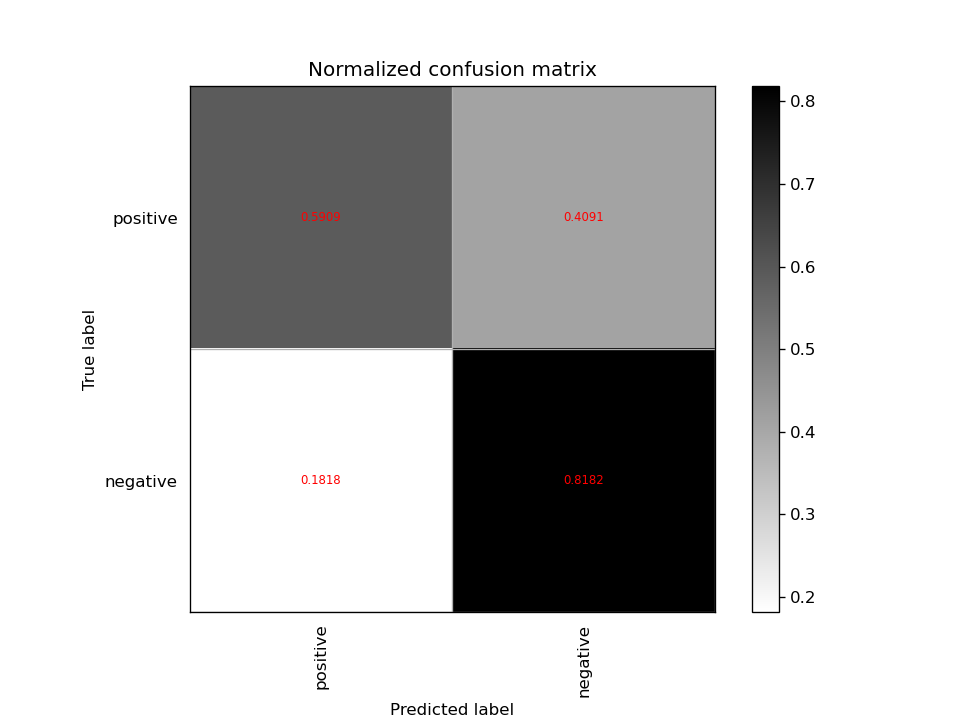

Supplement: Supplementary file 1 [file foods-10-00550-s001.zip › attachment/confusion matrix-SVM/214-1.png]

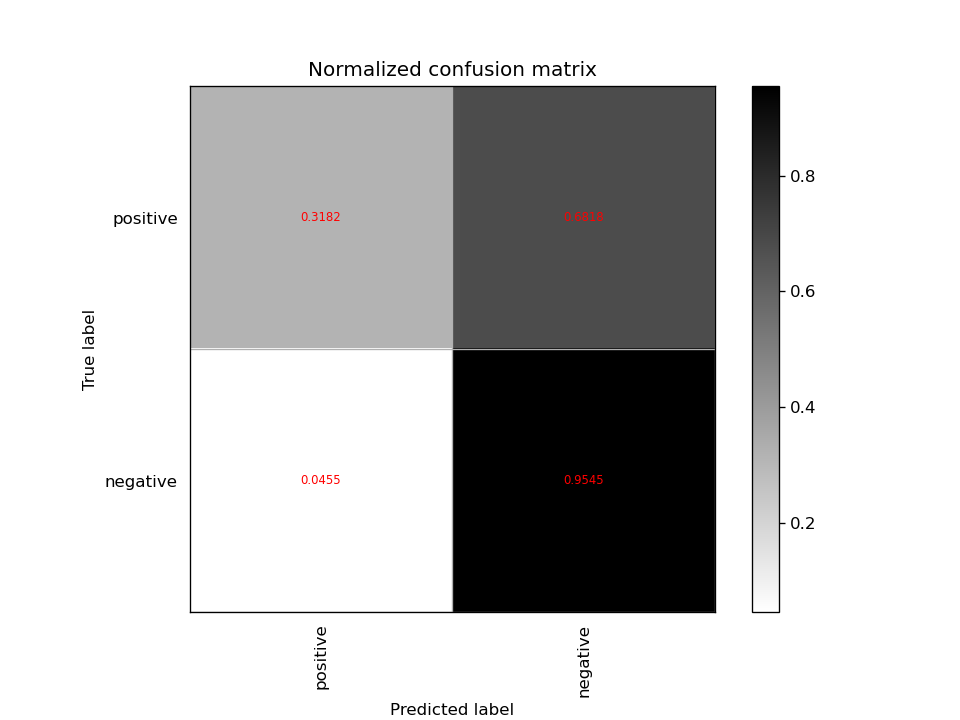

Supplement: Supplementary file 1 [file foods-10-00550-s001.zip › attachment/confusion matrix-SVM/214-2.png]

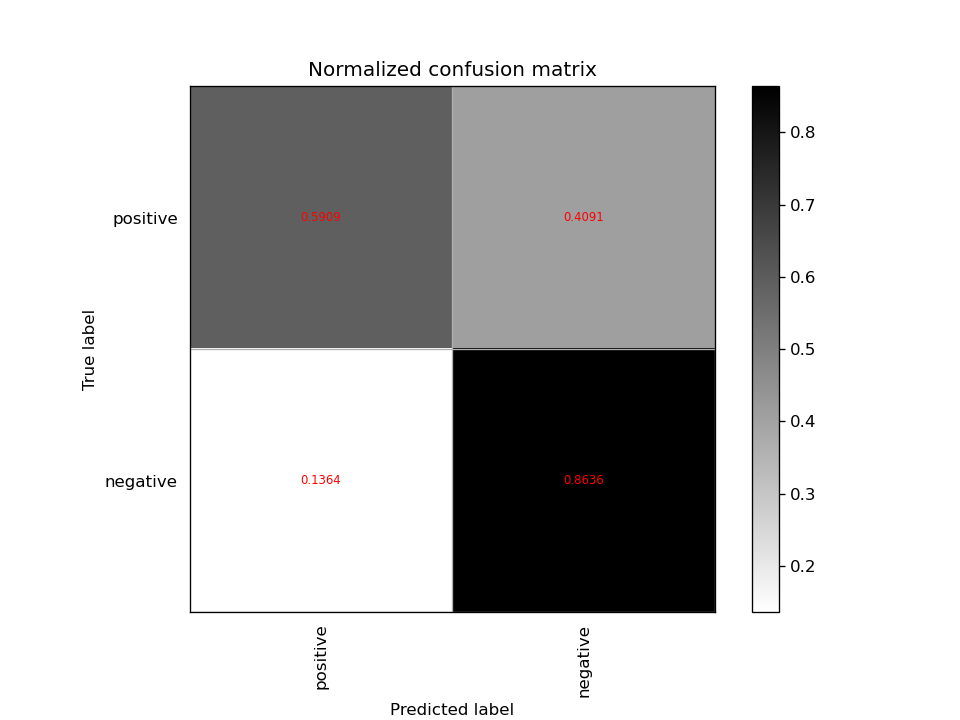

Supplement: Supplementary file 1 [file foods-10-00550-s001.zip › attachment/confusion matrix-SVM/214-3.png]

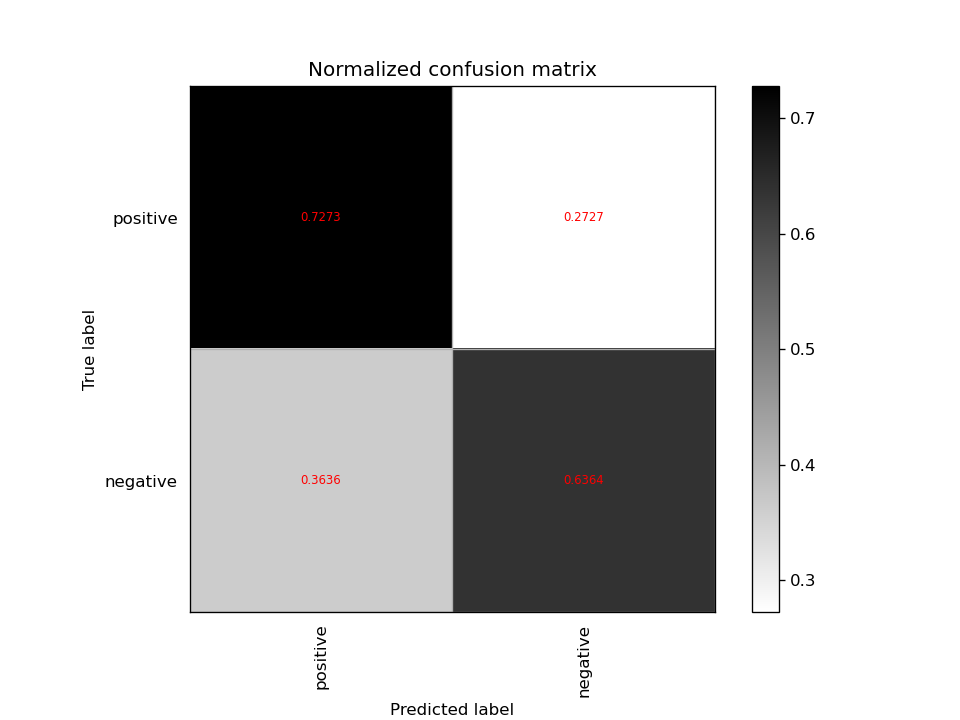

Supplement: Supplementary file 1 [file foods-10-00550-s001.zip › attachment/confusion matrix-SVM/214-4.png]

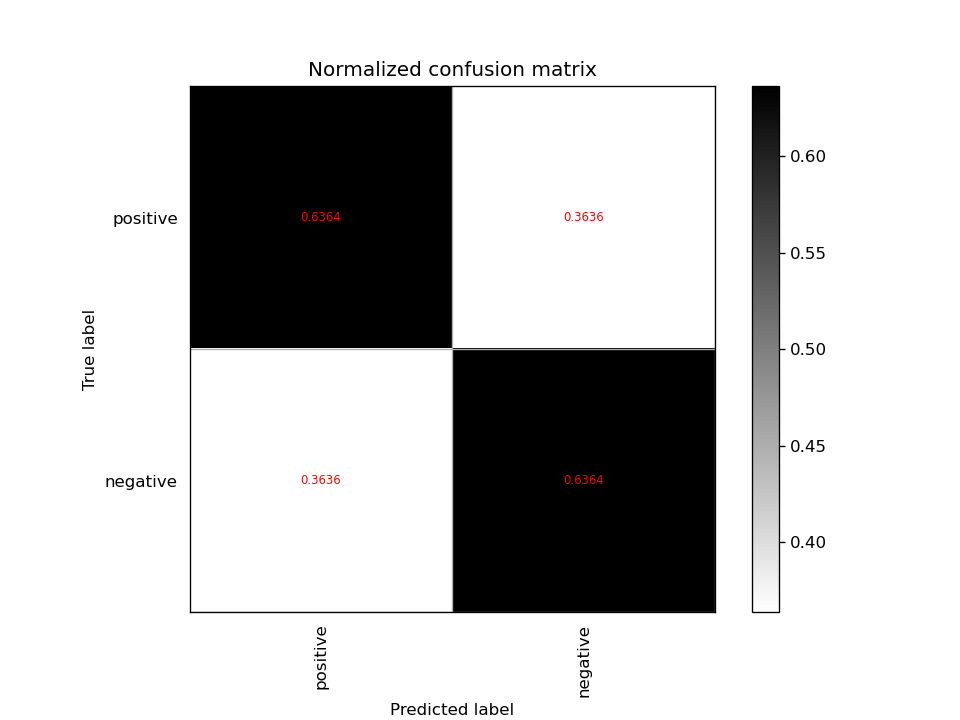

Supplement: Supplementary file 1 [file foods-10-00550-s001.zip › attachment/confusion matrix-SVM/214-5.png]

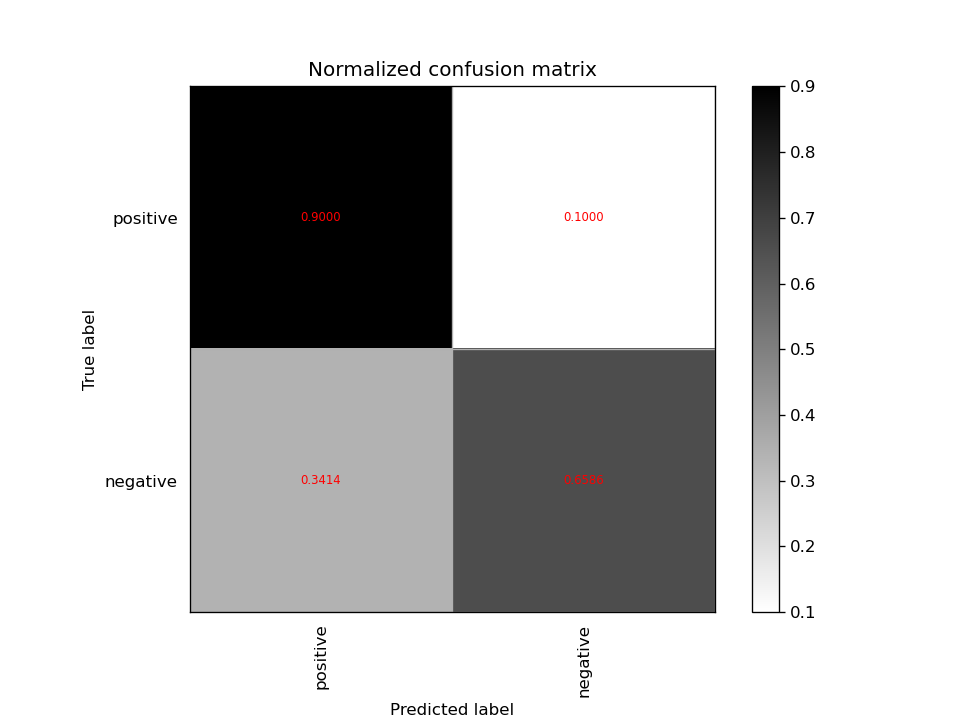

Supplement: Supplementary file 1 [file foods-10-00550-s001.zip › attachment/confusion matrix-SVM/3306-2.png]

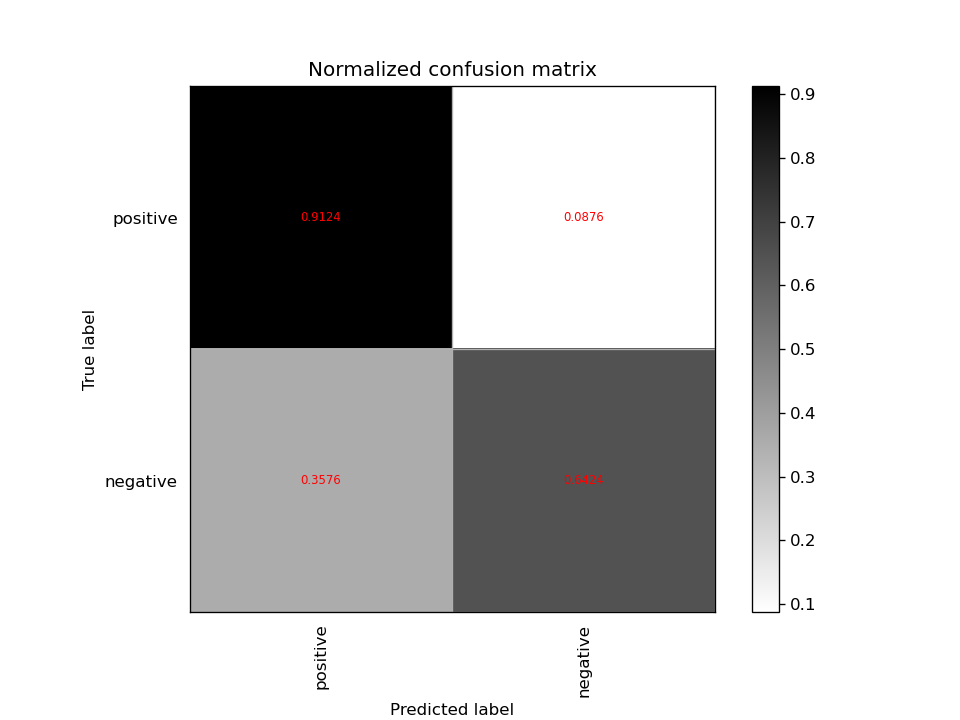

Supplement: Supplementary file 1 [file foods-10-00550-s001.zip › attachment/confusion matrix-SVM/3306-3.png]

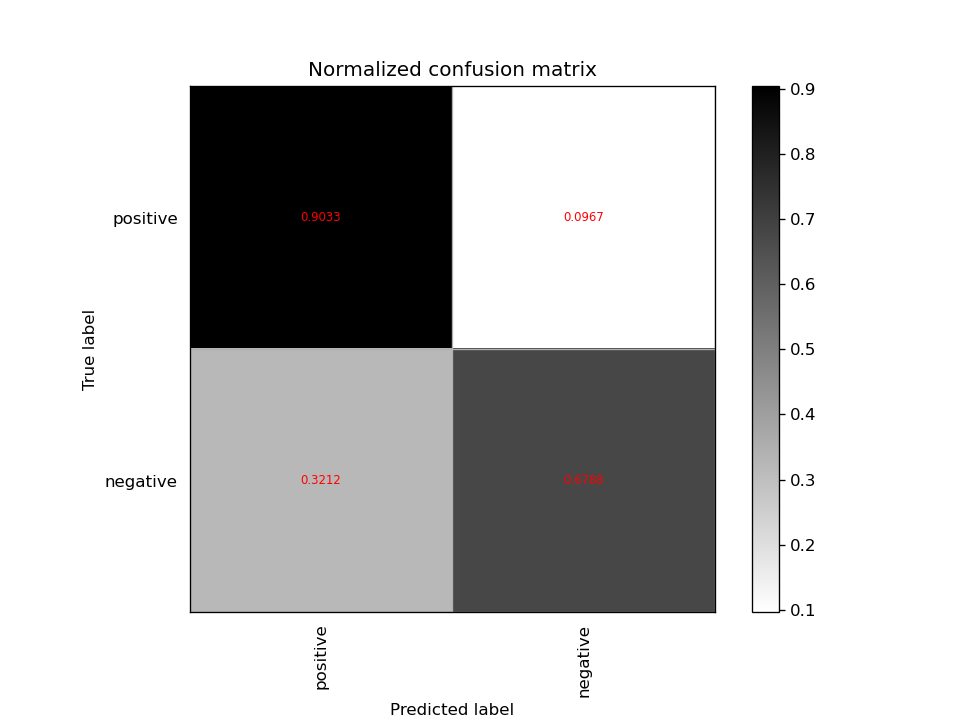

Supplement: Supplementary file 1 [file foods-10-00550-s001.zip › attachment/confusion matrix-SVM/3306-4.png]

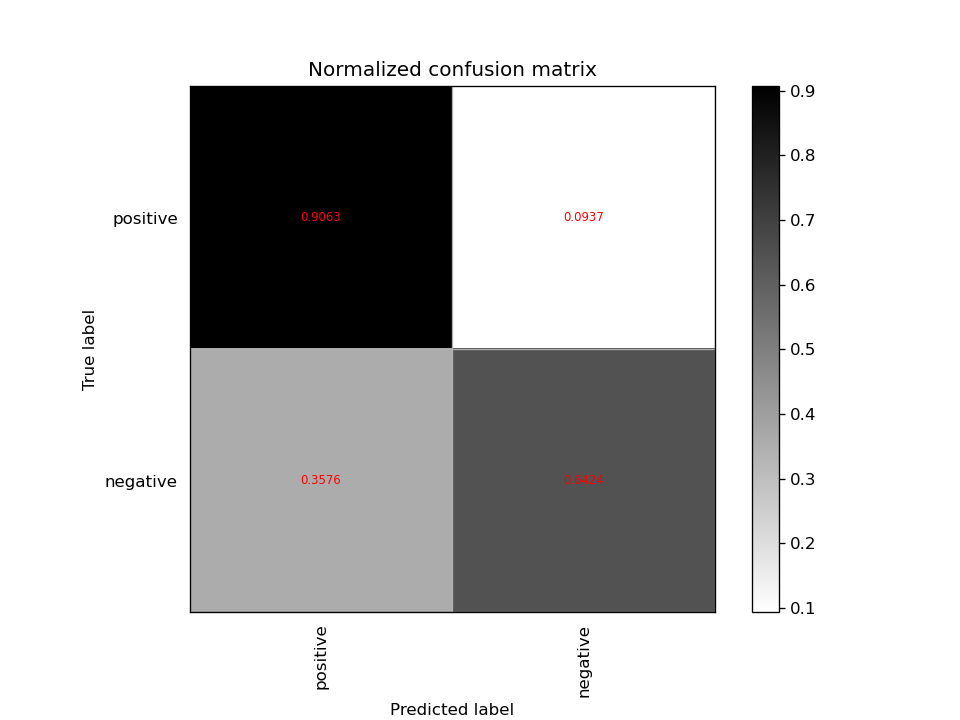

Supplement: Supplementary file 1 [file foods-10-00550-s001.zip › attachment/confusion matrix-SVM/3306-5.png]

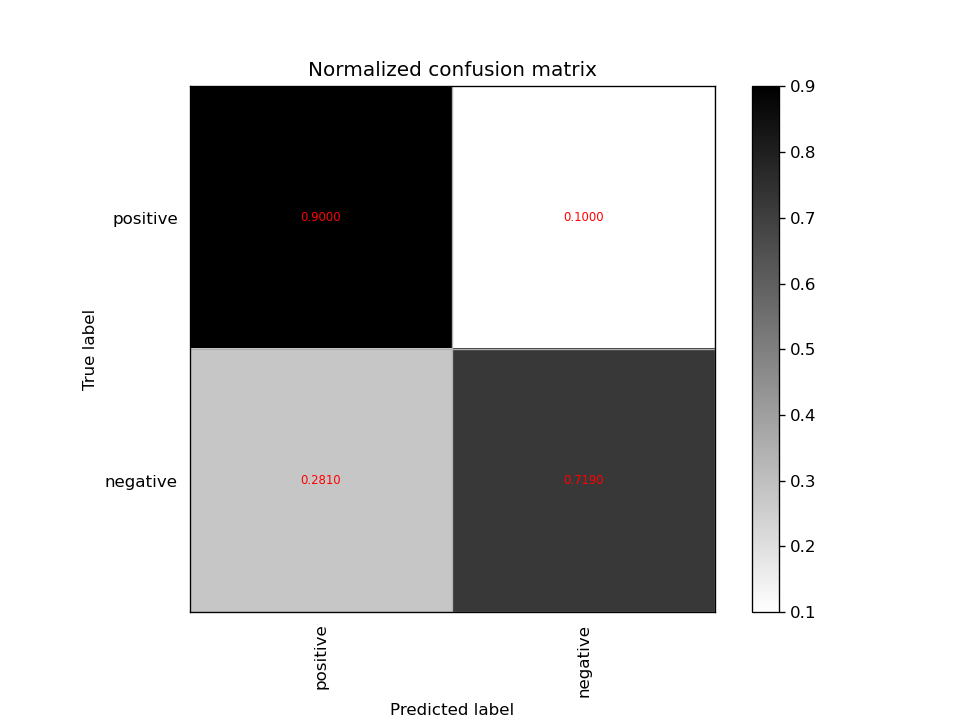

Supplement: Supplementary file 1 [file foods-10-00550-s001.zip › attachment/confusion matrix-SVM/3306.png]

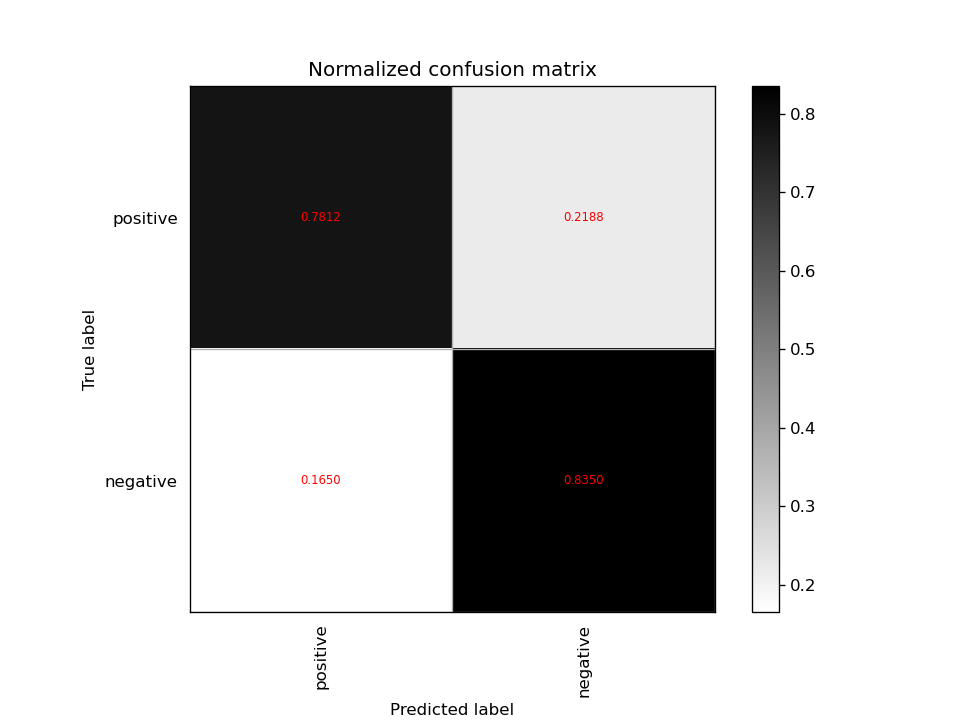

Supplement: Supplementary file 1 [file foods-10-00550-s001.zip › attachment/Confusion matrix-XGBoost/1378-1.png]

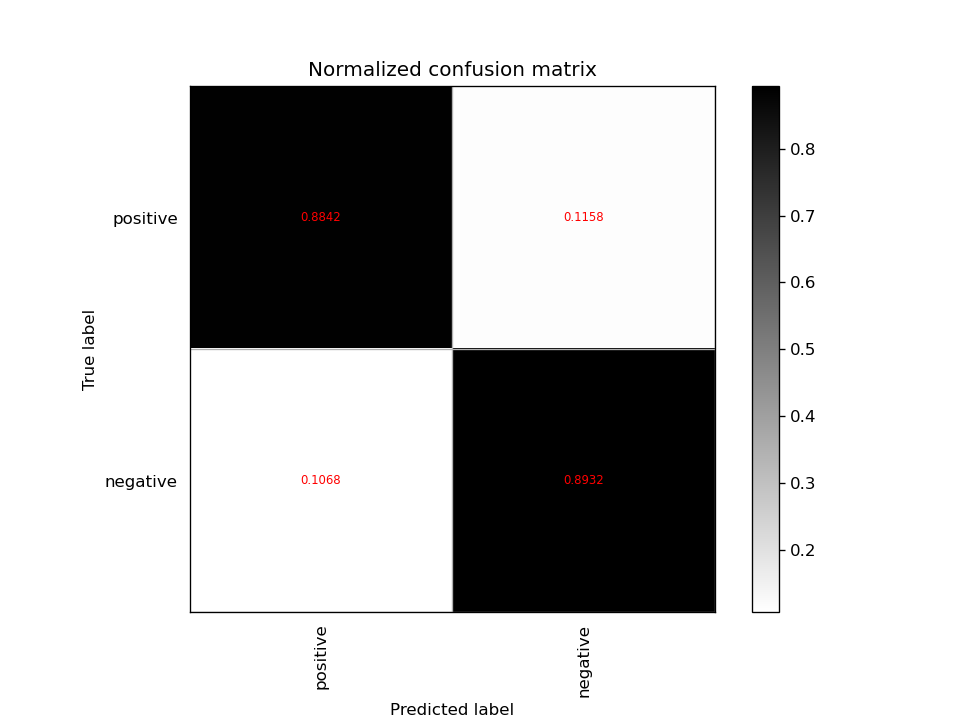

Supplement: Supplementary file 1 [file foods-10-00550-s001.zip › attachment/Confusion matrix-XGBoost/1378-2.png]

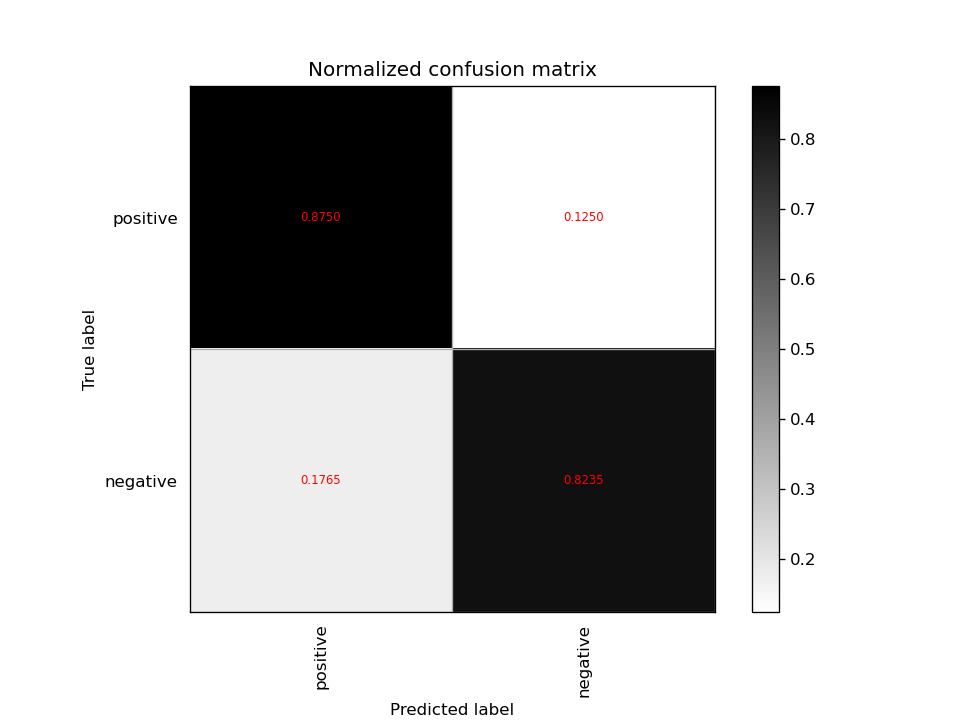

Supplement: Supplementary file 1 [file foods-10-00550-s001.zip › attachment/Confusion matrix-XGBoost/1378-3.png]

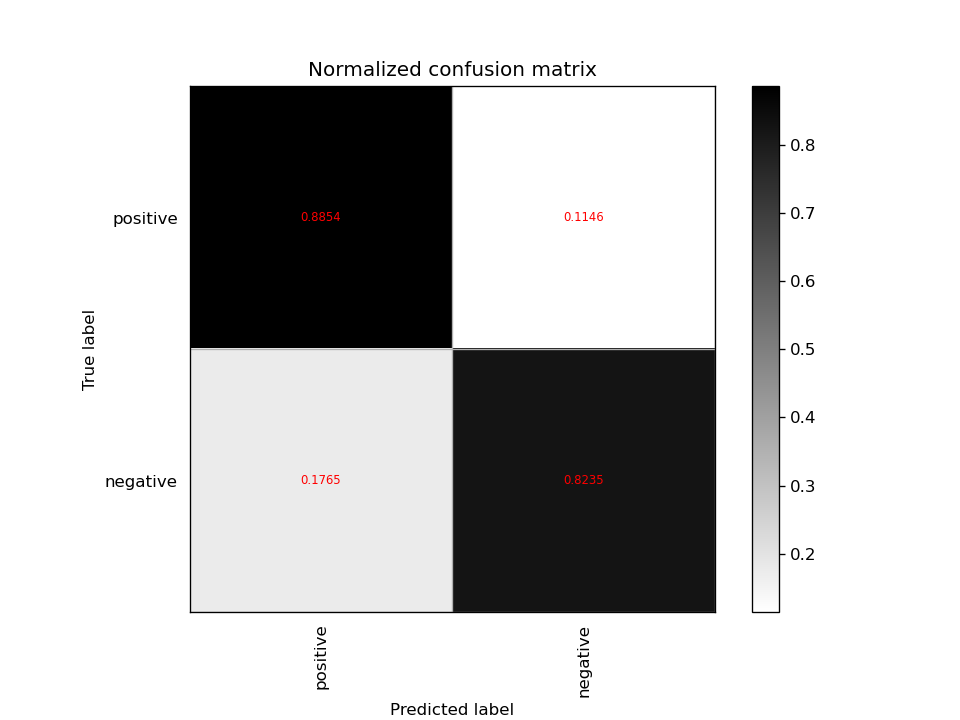

Supplement: Supplementary file 1 [file foods-10-00550-s001.zip › attachment/Confusion matrix-XGBoost/1378-4.png]

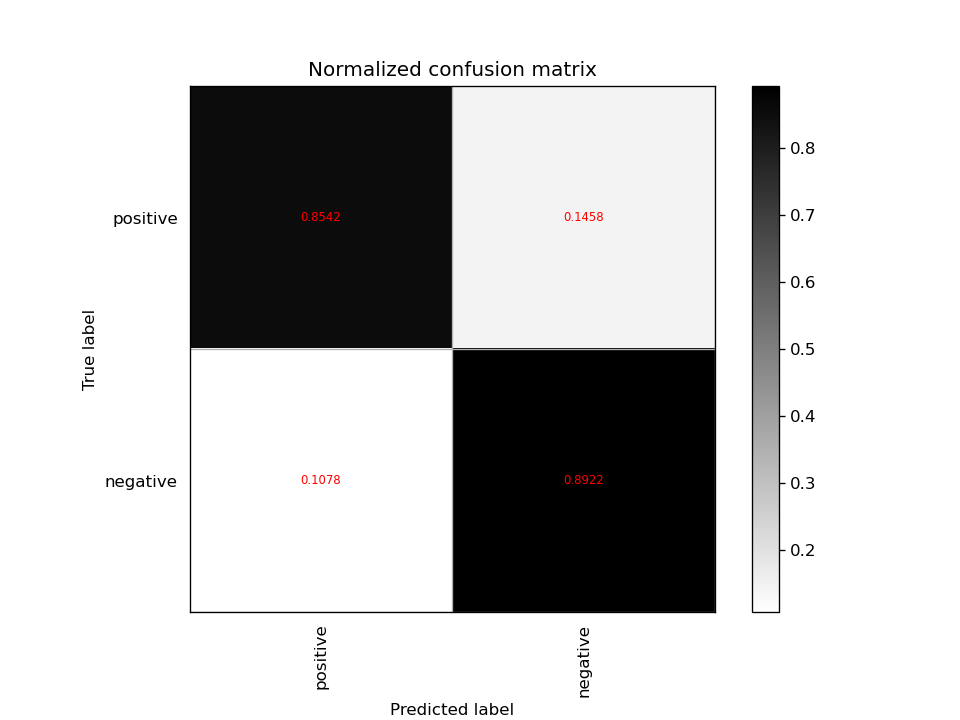

Supplement: Supplementary file 1 [file foods-10-00550-s001.zip › attachment/Confusion matrix-XGBoost/1378-5.png]

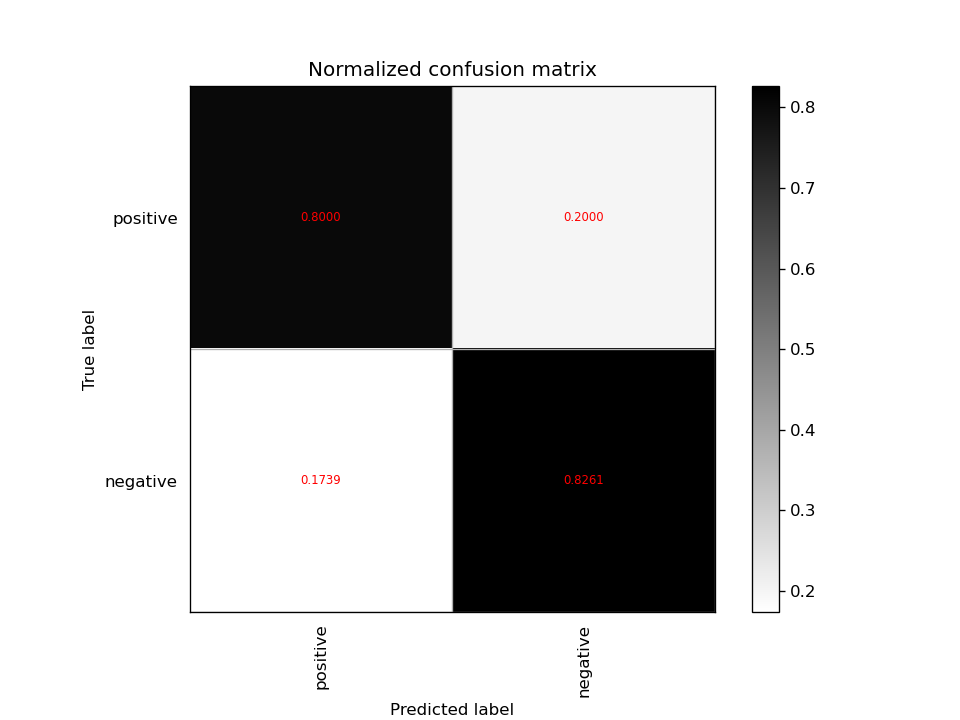

Supplement: Supplementary file 1 [file foods-10-00550-s001.zip › attachment/Confusion matrix-XGBoost/214-1.png]

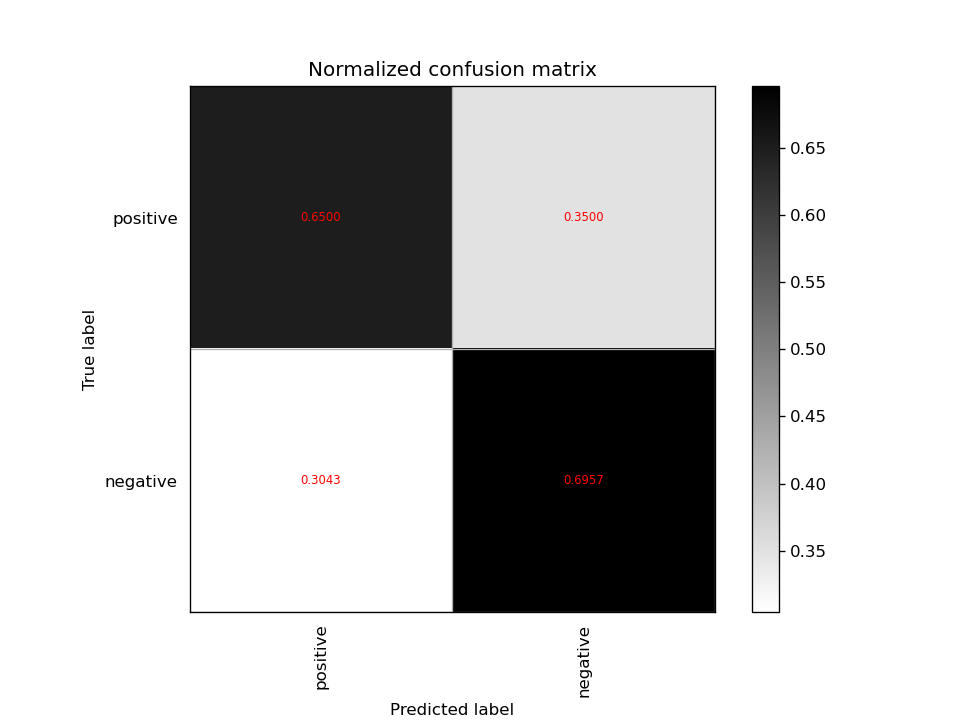

Supplement: Supplementary file 1 [file foods-10-00550-s001.zip › attachment/Confusion matrix-XGBoost/214-2.png]

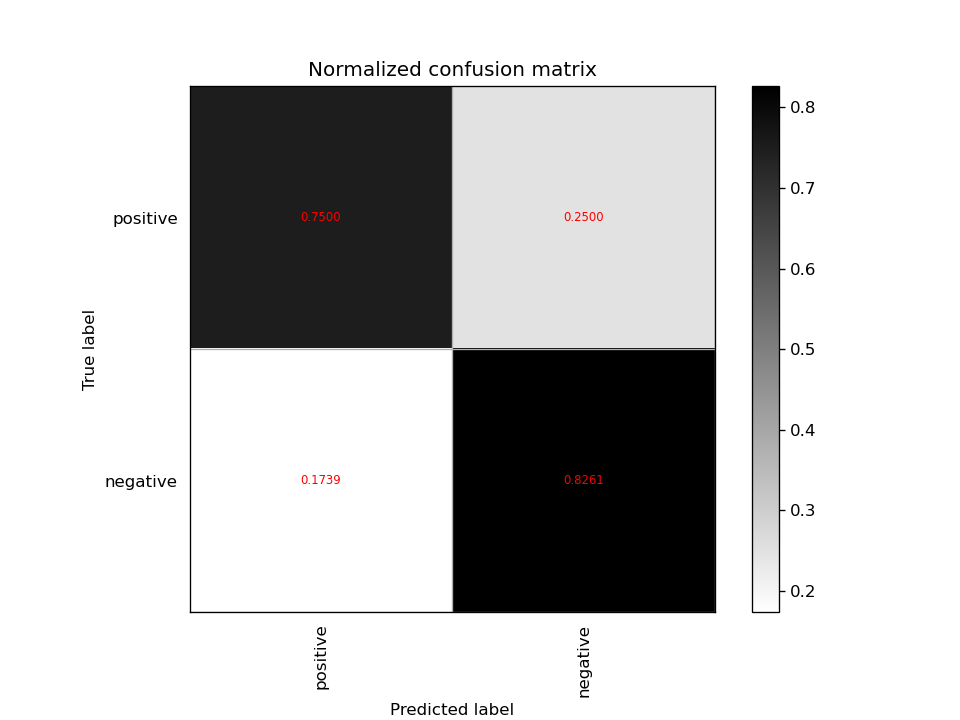

Supplement: Supplementary file 1 [file foods-10-00550-s001.zip › attachment/Confusion matrix-XGBoost/214-3.png]

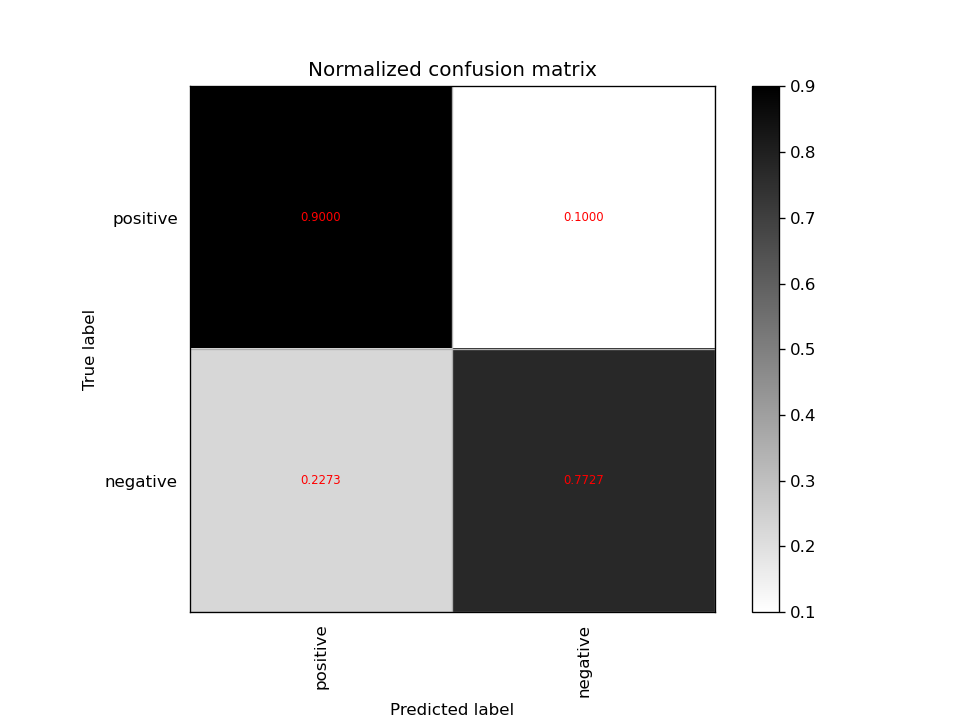

Supplement: Supplementary file 1 [file foods-10-00550-s001.zip › attachment/Confusion matrix-XGBoost/214-4.png]

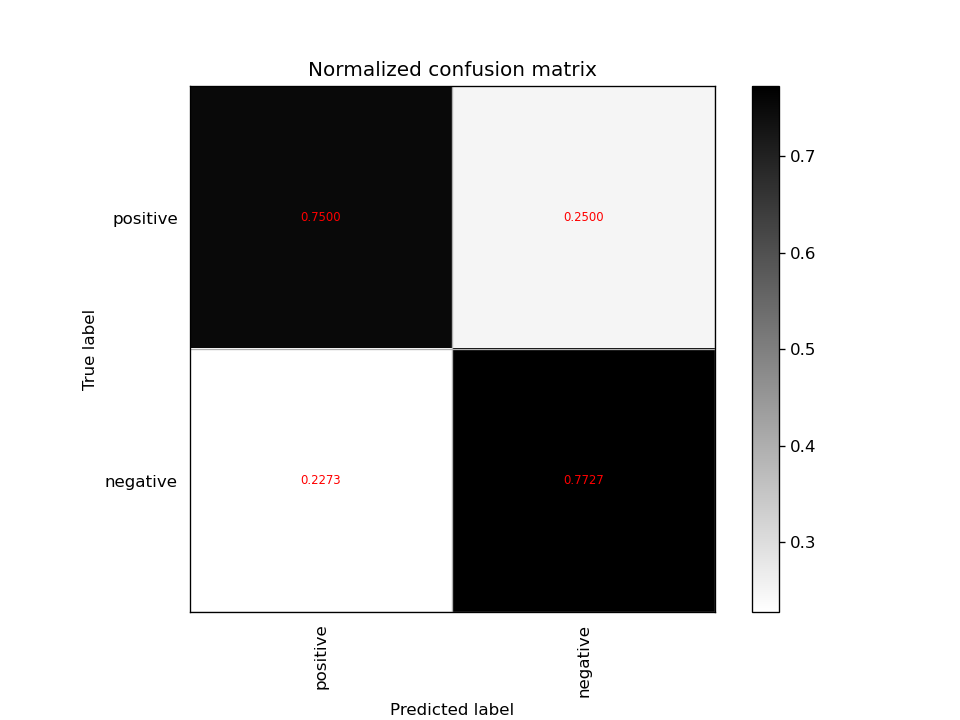

Supplement: Supplementary file 1 [file foods-10-00550-s001.zip › attachment/Confusion matrix-XGBoost/214-5.png]

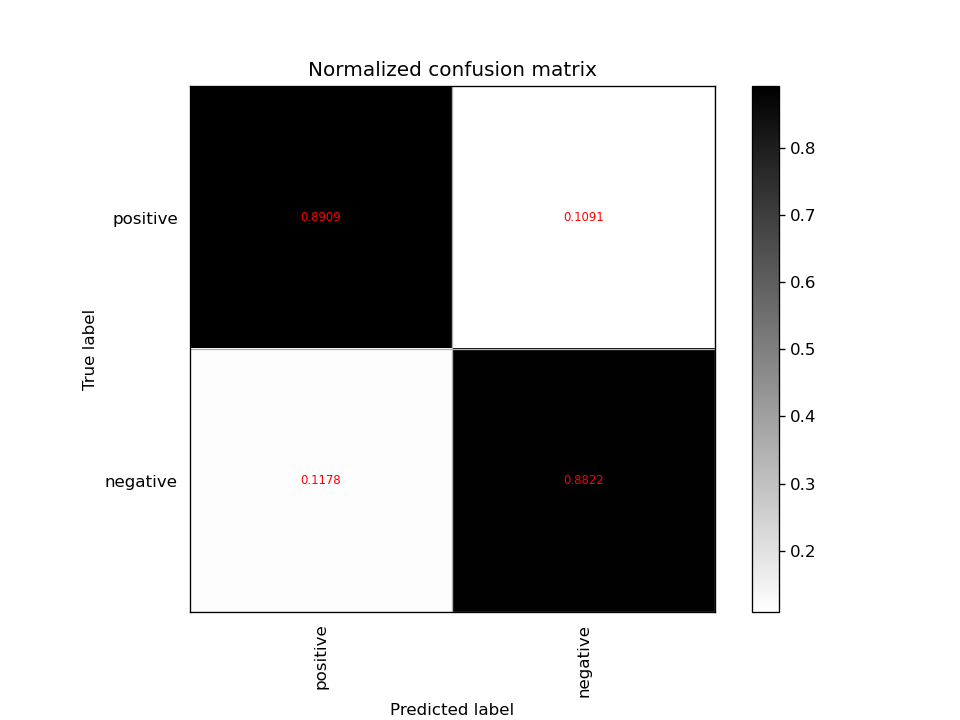

Supplement: Supplementary file 1 [file foods-10-00550-s001.zip › attachment/Confusion matrix-XGBoost/3306-1.png]

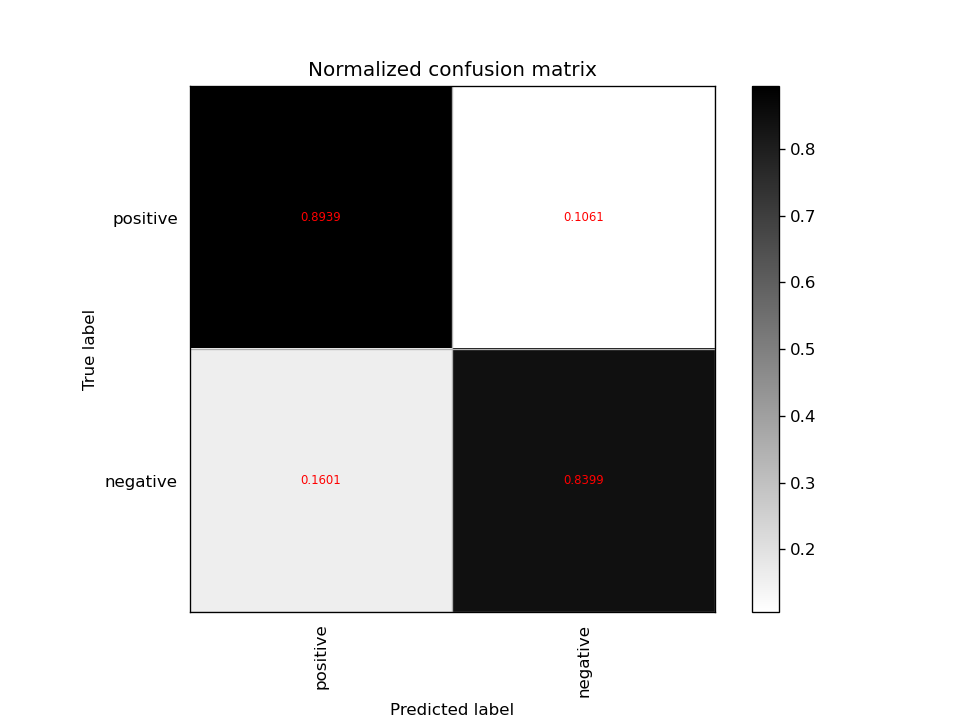

Supplement: Supplementary file 1 [file foods-10-00550-s001.zip › attachment/Confusion matrix-XGBoost/3306-2.png]

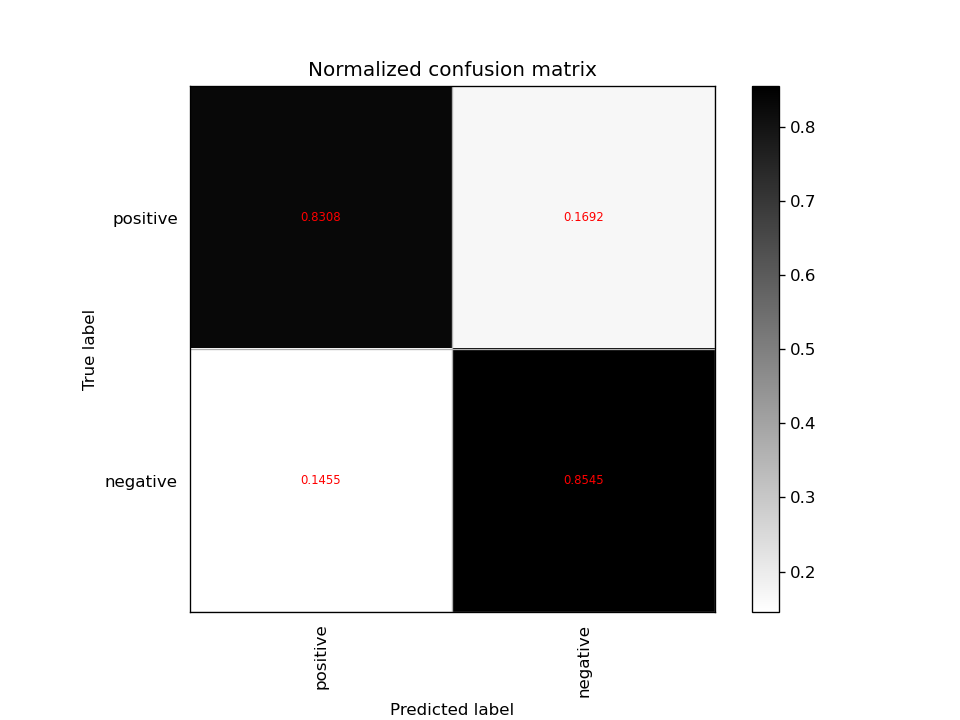

Supplement: Supplementary file 1 [file foods-10-00550-s001.zip › attachment/Confusion matrix-XGBoost/3306-3.png]

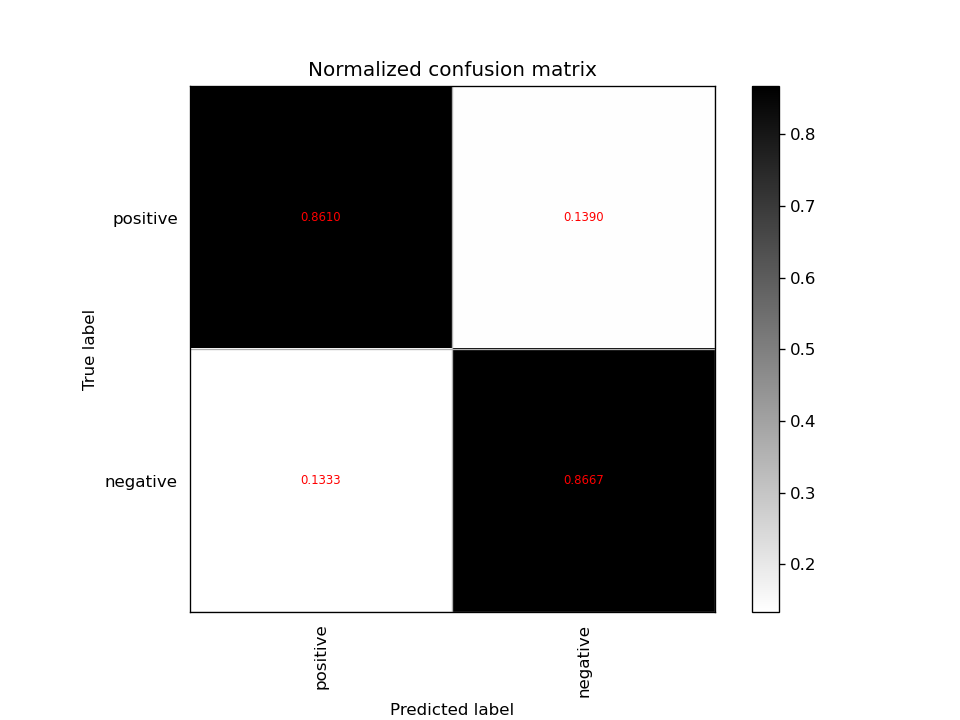

Supplement: Supplementary file 1 [file foods-10-00550-s001.zip › attachment/Confusion matrix-XGBoost/3306-4.png]

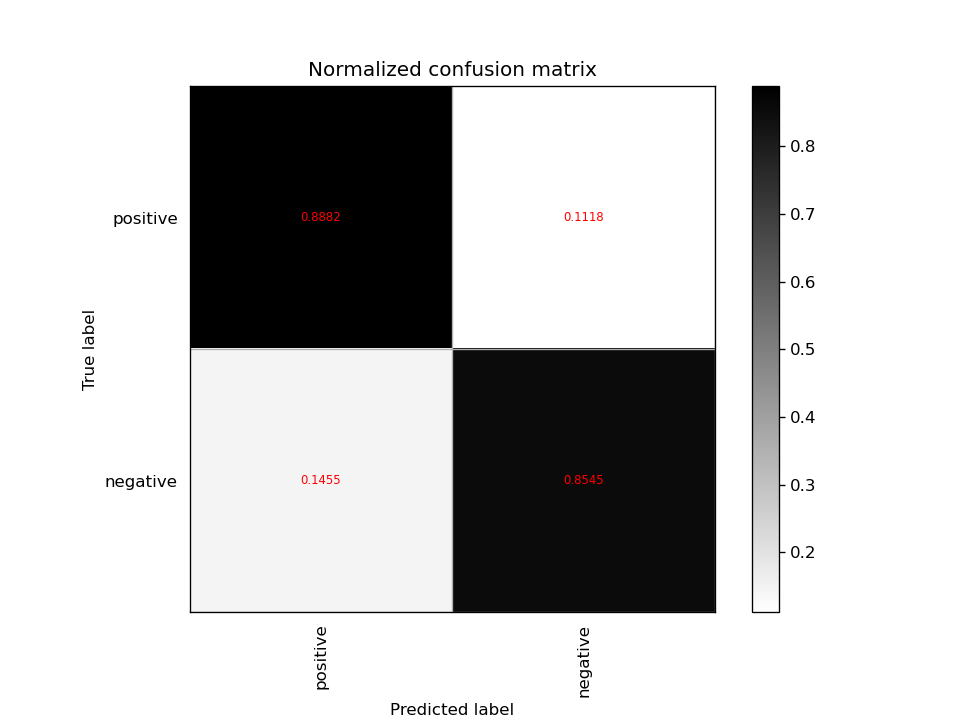

Supplement: Supplementary file 1 [file foods-10-00550-s001.zip › attachment/Confusion matrix-XGBoost/3306-5.png]
